# Reduction and Expansion in Microsporidian Genome Evolution: New Insights from Comparative Genomics

**DOI:** 10.1093/gbe/evt184

**Published:** 2013-11-19

**Authors:** Sirintra Nakjang, Tom A. Williams, Eva Heinz, Andrew K. Watson, Peter G. Foster, Kacper M. Sendra, Sarah E. Heaps, Robert P. Hirt, T. Martin Embley

**Affiliations:** ^1^Institute for Cell and Molecular Biosciences, The Medical School, Newcastle University, United Kingdom; ^2^Department of Biochemistry and Molecular Biology, Monash University, Clayton, Australia; ^3^Victorian Bioinformatics Consortium, Monash University, Clayton, Australia; ^4^Department of Life Sciences, Natural History Museum, London, United Kingdom; ^5^School of Mathematics and Statistics, Newcastle University, United Kingdom

**Keywords:** Microsporidia, intracellular parasites, evolution, genome reduction, gene duplication, novel gene families

## Abstract

Microsporidia are an abundant group of obligate intracellular parasites of other eukaryotes, including immunocompromised humans, but the molecular basis of their intracellular lifestyle and pathobiology are poorly understood. New genomes from a taxonomically broad range of microsporidians, complemented by published expression data, provide an opportunity for comparative analyses to identify conserved and lineage-specific patterns of microsporidian genome evolution that have underpinned this success. In this study, we infer that a dramatic bottleneck in the last common microsporidian ancestor (LCMA) left a small conserved core of genes that was subsequently embellished by gene family expansion driven by gene acquisition in different lineages. Novel expressed protein families represent a substantial fraction of sequenced microsporidian genomes and are significantly enriched for signals consistent with secretion or membrane location. Further evidence of selection is inferred from the gain and reciprocal loss of functional domains between paralogous genes, for example, affecting transport proteins. Gene expansions among transporter families preferentially affect those that are located on the plasma membrane of model organisms, consistent with recruitment to plug conserved gaps in microsporidian biosynthesis and metabolism. Core microsporidian genes shared with other eukaryotes are enriched in orthologs that, in yeast, are highly expressed, highly connected, and often essential, consistent with strong negative selection against further reduction of the conserved gene set since the LCMA. Our study reveals that microsporidian genome evolution is a highly dynamic process that has balanced constraint, reductive evolution, and genome expansion during adaptation to an extraordinarily successful obligate intracellular lifestyle.

## Introduction

Microsporidia are a diverse group of obligate intracellular parasites related to fungi that infect a wide range of eukaryotic hosts including both immunocompetent and immunocompromised patients (Didier and Weiss 2011; Vávra and Lukeš 2013). All species of Microsporidia share general features of their life cycle including environmental dispersal as resistant spores, invasion of new host cells using a specialized polar tube, and intracellular replication as meronts followed by intracellular differentiation into spores (Vávra and Lukeš 2013). Infection produces a variety of disease symptoms, depending on the Microsporidia species and host, but a general understanding of the infection process and the underpinning interactions between parasite and host at the molecular level is still at an early stage (Delbac and Polonais 2008; Troemel et al. 2008; Tsaousis et al. 2008; Vávra and Lukeš 2013). This is due in part to the difficulties of studying obligate intracellular parasites that generally cannot be maintained in tissue culture and for which there are no well-established tools for genetic manipulation (Paldi et al. 2010).

Analysis of microsporidian genomes can provide a comparative framework for understanding how microsporidians have become so successful as parasites and help to guide, and perhaps prioritize, experimental work. The microsporidian genomes for which data are available vary in size by an order of magnitude (2.3–24 Mb) (Corradi et al. 2009, 2010), but these large differences appear to reflect differences in gene density caused by the variable presence of transposable and repetitive elements rather than large variation in gene number (Heinz et al. 2012; Peyretaillade et al. 2012). The number of predicted protein-coding genes varies over a much smaller range of approximately 1,750–3,266 genes, depending on the species and method of analysis (Cornman et al. 2009; Cuomo et al. 2012; Heinz et al. 2012; Peyretaillade et al. 2012). Although some of these gene differences are of known functional significance, for example, the presence or absence of glycolytic enzymes (Keeling et al. 2010; Peyretaillade et al. 2012), or variation in copy number of the functionally important surface-located nucleotide transport proteins (Tsaousis et al. 2008; Cuomo et al. 2012), most differences involve hypothetical genes of unknown function (Cuomo et al. 2012; Heinz et al. 2012; Peyretaillade et al. 2012). Examples of lineage-specific gene family expansions include an expanded family of leucine-rich repeat (LRR)-containing proteins in the genome of *Trachipleistophora hominis* (Heinz et al. 2012) and an expanded gene family called interB in microsporidian species belonging to the genera *Encephalitozoon, Vittaforma,* and *Anncaliia* (Dia et al. 2007). The functional importance of these expanded protein families is currently unknown.

In this study, we used a variety of methods to identify and compare the variable and conserved gene content of 11 published genomes spanning a broad sample of microsporidian diversity (Vossbrinck and Debrunner-Vossbrinck 2005; Troemel et al. 2008). Genes common to all microsporidians are especially interesting because they should include the genes that are essential for completing core features of the parasite life cycle including any general mechanisms used for host exploitation. We sampled species with compacted genomes and species with larger, less gene-dense, genomes, and integrated our findings with data from published expression and proteomics studies (Brosson et al. 2006; Cuomo et al. 2012; Heinz et al. 2012; Grisdale et al. 2013). Our analyses demonstrate that microsporidian genome evolution has been more dynamic than hitherto appreciated. An early bottleneck caused by massive gene loss left a highly reduced ancestral proteome which included a core set of genes whose orthologs in yeast are among the most essential, highly connected, and highly expressed genes; factors that help to explain their retention and resistance to further gene loss. The reduced metabolism inferred by the core set has been complemented by expansion of predicted surface-located transporter families to import essential substrates from the host cytoplasm. Additional expansion and innovation has affected a family of putative mechanosensitive ion channel (MscS) proteins, members of the CAP family of proteins, zinc metalloproteases, and a set of novel gene families that are enriched for potentially secreted or surface-located proteins. The conservation among microsporidians of these expanded families, against a strong background of reductive evolution, suggests that they mediate conserved features of host–parasite interactions.

## Materials and Methods

### Genome Sequence Data

Our comparative analysis included 11 microsporidian, 3 animal, and 7 fungal genomes. The following microsporidian genomes were included in the analysis: *Encephalitozoon intestinalis* ATCC 50506 (Corradi et al. 2010) (RefSeq: NC_014415-NC_014425); *E. cuniculi* GB-M1 (Katinka et al. 2001) (RefSeq: NC_003229-NC003238, NC_003242); *E. hellem* ATCC50504 (Pombert et al. 2012) (MicrosporidiaDB, downloaded August 3, 2012); *E. romaleae* SJ-2008 (Pombert et al. 2012) (GenBank: CP003518–CP003530); *Nosema ceranae* BRL01 (Cornman et al. 2009) (RefSeq: NW_003308796-NW_003314260); *T. hominis* (Heinz et al. 2012) (GenBank: JH993798–JH994107); *Vavraia culicis floridensis, Vittaforma corneae* ATCC 50505, *Nematocida parisii* ERTm1, *N. parisii* ERTm3, and *Nematocida sp1* ERTm2 (Cuomo et al. 2012). The latter five microsporidian genomes were derived from the Broad Institute of Harvard and Massachusetts Institute of Technology (Microsporidia Comparative Sequencing Project, http://www.broadinstitute.org/, last accessed November 29, 2013) (downloaded August 3, 2012). See supplementary data, Supplementary Material online, for details of the fungal and animal genomes that were included in the analysis.

### Gene Family Construction

To construct homologous gene families, we classified all available protein sequences from 11 microsporidian, 7 fungal, and 3 animal genomes into clusters of homologs using Markov Clustering (MCL) (Enright et al. 2002) with inflation rate of *I* = 1.2. This inflation rate yielded the best *F*-measurement value of 0.40. The procedure for selecting inflation rate for MCL is the same procedure as used in Carman and Han (2011) and Heinz et al. (2012). BlastP (Altschul et al. 1990) with a low complexity mask was used for sequence similarity searches. The input values for MCL were *e* values (cutoff ≤ 10^−^^5^) from all-against-all BlastP searches with an alignment length cutoff as follows: 0.5 for both query and target sequences if both sequences are from nonmicrosporidian genomes, 0.5 for either query or target sequence if they both are from microsporidian genomes, and 0.45 for microsporidian sequence if searches against sequences from nonmicrosporidian genomes. Less strict alignment length cut-off values were applied to microsporidian protein sequences, because they are often shorter and more divergent than their homologs in fungal or animal genomes.

Microsporidian sequences are often highly divergent from their fungal and animal homologs, and therefore the Blast cut-off values described above are too strict for some of these divergent sequences to form a cluster with homologs from outside the Microsporidia. As a result, an exclusive cluster is formed that contains only microsporidian sequences. To reconcile fungal and animal homologs of these microsporidian sequences, we merged microsporidian-only clusters with another cluster if at least one member from each of the two clusters were reciprocal best BlastP hits, that is, two sequences, one from each cluster, that are each other’s respective top BlastP hit (*e *value cutoff: 0.001).

A file with all protein families containing microsporidian members and listing the accession numbers of the individual proteins making up these families can be downloaded from: http://figshare.com/articles/microsporidian_protein_families/834937 (last accessed November 29, 2013).

### Ancestral Reconstruction of Gene Families during Microsporidian Evolution

**Fig. 1.—:**
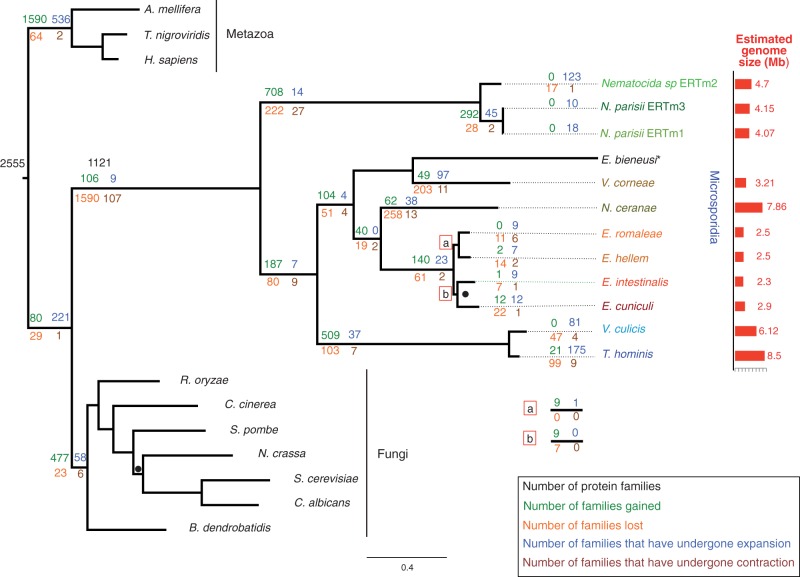
Gain, loss, expansion, and contraction of protein families during the evolution of microsporidian genomes. The history of gene content evolution was estimated using a phylogenetic birth–death model implemented in Count (Csurös 2010). Gains (green), losses (orange), expansions (blue), and contractions (brown) were mapped onto the concatenated protein phylogeny. Following a massive ancestral reduction, gain and expansion outweigh loss and contraction on some branches of the microsporidian tree. The phylogeny of the Microsporidia and their opisthokont relatives was inferred from a concatenation of 23 congruent, single-copy orthologous genes conserved across the 21 genomes analyzed in this study. * denotes an additional microsporidian, *Enterocytozoon bieneusi*, that is excluded from the gene content evolution analysis because of substantial genomic contamination. The tree was built with the CAT model in PhyloBayes (Lartillot et al. 2009); branch lengths are proportional to substitutions per site, as indicated by the scale bar. Based on previous work (Hirt et al. 1999), the tree was rooted on the branch leading to the Metazoa. All nodes have posterior probabilities of more than 0.95, unless denoted by black dots (nodes with posterior probabilities between 0.85 and 0.90).

Reconstruction of gene gain and loss during microsporidian evolution was performed using the phylogenetic gain–loss-duplication model employed in the software package Count (Csurös 2010). The reconstruction takes into account the number of paralogs in each genome for all identified gene families, and the concatenated protein species tree (fig. 1) was used as the reference tree. The model was fit using the maximum-likelihood procedure implemented in Count, with the default null model as a starting point, and with gain, loss, and duplicate rates estimated for all branches of the tree. Rates of gain, loss, and duplication were drawn from a discretized gamma distribution with four categories. The ML optimization was set to stop when one of these convergence criteria is met: 1) after the maximum of 50 rounds or 2) when the log-likelihood in two consecutive rounds changes by less than a convergence threshold of 0.1.

### Identification of Gene Families Expanded in the Microsporidia

Phylogenies for each gene family were used to identify families that have undergone duplication during the evolution of Microsporidia. We first constructed phylogenetic gene trees of families that contain at least two sequences from one microsporidian genome and at least two sequences from other representative opisthokont genomes included in the analysis. Protein sequences in each family were aligned using two different methods, MAFFT (Katoh et al. 2002) and MUSCLE (Edgar 2004). A consensus alignment was then generated using T-Coffee (Notredame et al. 2000), and the resulting alignment was trimmed using trimAl (Capella-Gutiérrez et al. 2009) with the automated1 option. Phylogenetic trees based on maximum likelihood (ML) were constructed using RAxML (Stamatakis et al. 2005) with the PROTCATLG model and 100 bootstrap replicates. Each gene tree was then processed through an in-house script that identified well-supported (bootstrap value >80%) duplications within the Microsporidia lineage. A gene tree was identified as having a family expansion within the microsporidian lineage if two or more copies of the gene from one microsporidian genome were found clustered within a monophyletic clade that only contain microsporidian sequences.

### Phylogenetic Analysis of Gene Families

All gene trees present in the figures were constructed based on Bayesian analysis using PhyloBayes (Lartillot et al. 2009). The CAT60 model implemented in PhyloBayes was used on trimmed multiple sequence alignment. The trimmed alignments were generated using the same procedure as described earlier. For each gene family, a consensus tree of trees generated from two independent Markov chain Monte Carlo chains was constructed. Convergence was assessed using the criteria of 1) a maximum bipartition discrepancy (maxdiff) < 0.1 and 2) a minimum effective size > 100 for all sampled parameters. All alignments used to estimate the phylogenies are available upon request.

### Protein Sequence Feature Prediction

SignalP 4.0 (Petersen et al. 2011) was used to predict N-terminal secretory signals. The presence of alpha-helix transmembrane domains (TMDs) was determined using TMHMM 2.0 (Krogh et al. 2001). For identification of protein domains, protein sequences were analyzed using InterproScan 4.8 (Zdobnov and Apweiler 2001) searching against PFAM (Finn et al. 2009) database. Intrinsically disordered regions were identified based on the consensus results from multiple prediction tools used in MESSA (Cong and Grishin 2012).

### Analysis of Gene Order Conservation

To quantify the level of synteny between pairs of genomes, we calculated the proportion of single-copy orthologous gene pairs that are located next to each other in both genomes. To avoid an underestimate of the level of synteny, genes located at contig ends were not included in our calculations. InParanoid (Alexeyenko et al. 2006) was used to generate clusters of orthologous and paralogous genes from a pair of given genomes. An in-house script was used to select clusters that do not contain paralogs. The resulting clusters therefore containing single-copy orthologous gene pairs from two genomes. OrthoCluster (Zeng et al. 2008) was then used to measure the level of gene order conservation between two genomes. The option -l 2 was used, so that only two genes that are adjacent to each other in both genomes were classified as syntenic.

### Regression Analysis

All the regression models discussed in the main text were fit using R (R Development Core Team 2013). We used a generalized linear model to evaluate the relationship between retention in the microsporidian core gene set (modeled as a binomial response) and expression level (protein molecules/cell) (Ghaemmaghami et al. 2003), number of interactions (Cherry et al. 2012), and essentiality in yeast (Cherry et al. 2012) (modeled as fixed effects). The model was fit using the glm function in R. To estimate the effect of core membership on expression level, we fit a linear mixed-effects model in a Bayesian framework using OpenBUGS (Lunn et al. 2009) and the “BRugs” R package to avoid complications with ML parameter estimation for unbalanced data (e.g., differing numbers of genes per gene family); in this analysis, estimates are posterior medians and effects are judged to be significant when the 95% Bayesian credible interval does not overlap zero. The reported results were robust to the choice of prior. We investigated the relationship between log-transformed *E. cuniculi* expression levels (FKPM values) (Grisdale et al. 2013) and core status (binary fixed effect), time point (continuous covariate), and nested random effects for gene family in core status and gene in gene family. To compare the retention of genes of archaeal and bacterial origin in the core microsporidian proteome, we used the reference list of archaeal and bacterial genes in eukaryotes from Cotton and McInerney (2010). We then performed a Fisher’s exact test on a contingency table of bacterial and archaeal genes in *Saccharomyces cerevisiae* which have been retained or lost in Microsporidia.

### The Relationship between Synteny, Protein Identity, and Proportion of Intergenic DNA

The generalized linear model was fit using R (http://www.r-project.org, last accessed November 29, 2013). Since the effect of protein sequence identity and intergenic DNA on synteny is not necessarily linear, we also investigated the relationships between these variables using nonparametric approaches, including a generalized additive model (gam) and Spearman’s rank correlation. These methods agreed with the linear model on the direction and significance of the effects reported, suggesting our results are robust to the approach used.

## Results and Discussion

### Widespread Gene Family Expansion After a Bottleneck in the Last Common Ancestor of Microsporidia

To provide a phylogenetic framework for our comparative analyses of microsporidian genome content, we built a Bayesian phylogenetic tree for 11 microsporidian genomes and a selection of opisthokont outgroups (fig. 1) using the CAT model in PhyloBayes (Lartillot et al. 2009); this phylogenetic model is particularly appropriate for the analysis of microsporidian sequences because it is less susceptible than simpler single-matrix models to long branch attraction (Lartillot et al. 2007), an artifact which has traditionally plagued microsporidian phylogenies (Hirt et al. 1999). The microsporidian genomes sampled include four *Encephalitozoon* species (*E. cuniculi*, *E. intestinalis*, *E. hellem*, and *E. romaleae*), three *Nematocida* isolates (*N. parisii* ERTm1 and ERTm3, *Nematocida* sp1 ERTm2), as well as *Nosema ceranae* BRL01, *Vi. corneae* ATCC 50505, *Vavraia culicis floridensis*, and *T. hominis* (supplementary table S1, Supplementary Material online). The tree was constructed based on a concatenation of 23 single-copy orthologous protein-coding genes shared among all 22 genomes included in the analysis that were first determined to contain congruent phylogenetic signal using a hierarchical likelihood ratio test (Leigh et al. 2008). An additional microsporidian, *Enterocytozoon bieneusi* (Akiyoshi et al. 2009), was included in this tree but excluded from subsequent analyses of gene content evolution because of substantial genomic contamination (Heinz et al. 2012; Peyretaillade et al. 2012). The published set of genomes that we analyzed comprises a broad sample of known microsporidian diversity, including representatives from Clades II–IV (Troemel et al. 2008).

We clustered all available protein sequences from the 22 species into families, each containing at least two homologous sequences from one or more genomes (see Materials and Methods). To investigate the evolution of microsporidian genomic diversity, we applied a phylogenetic gain–loss-duplication model implemented in the program Count (Csurös 2010) on the inferred phylogeny to estimate the number of gains and losses as well as the expansion and contraction of protein families during the evolution of Microsporidia (fig. 1). This probabilistic, ML-based approach has several advantages over traditional Dollo parsimony reconstructions of ancestral gene content: not only can changes in family size be inferred but also ancestral proteome sizes are estimated by summing over the probability of presence for each family, allowing uncertainty in the history of individual gene families to be accommodated in a natural way (Csurös and Miklós 2009). Using this method, the ancestor of the microsporidian species sampled was inferred to encode 1,121 protein families (fig. 1).

Our reconstruction is consistent with an earlier inference of both extensive gain and loss of gene families in the ancestral microsporidian based on parsimony analysis (Heinz et al. 2012) but further implies major, lineage-specific gene content evolution following divergence from the last common microsporidian ancestor (LCMA) (fig. 1). In addition to methodological improvements, our new reconstruction of gene content evolution is more complete because the availability of *Nematocida* spp. provides an outgroup to the previously analyzed genomes, enabling us to push our ancestral reconstruction further back toward the common ancestor of the Microsporidia as a whole. The bottleneck at the LCMA (in which 1,590 families are predicted to have been lost) was followed by sustained expansion of gene families in many microsporidian lineages, both through increases in family size and gain of entirely new families (fig. 1). A comparison of the estimated numbers of gains, losses, expansions, and contractions on the branches of the tree in figure 1 suggests that the rates of these processes vary among extant microsporidian lineages; for instance, the *T. hominis/V. culicis* and *Nematocida* clades appear to have gained many more gene families than the *Encephalitozoon* clade. To test this hypothesis, we compared the fit of the free-ratio model plotted in figure 1 to a model in which all microsporidian lineages were constrained to evolve under the same gain–loss ratio. The fit of this more restrictive model was much worse (*P* = 2.34 × 10^−^^192^, likelihood ratio test), implying that the tempo of gene content evolution varies significantly across the microsporidian radiation. In the case of the *Encephalitozoon* spp., this low rate of gene family gain is associated with the most compact microsporidian genomes known.

New families may represent acquisitions by horizontal gene transfer (HGT), ancestral families that have diverged so far that their relationships to other sequences have been obscured, or de novo formation of new genes—that is, the evolution of genes from previously noncoding sequence (Carvunis et al. 2012). To distinguish between these possibilities and to understand the contribution of new and expanded protein families to microsporidian biology, we performed a detailed bioinformatic characterization of both lineage-specific and conserved core protein families.

### Novel, Microsporidian-Specific Protein Families Are Likely to Play an Important Role in Host–Parasite Interactions

**Fig. 2.—:**
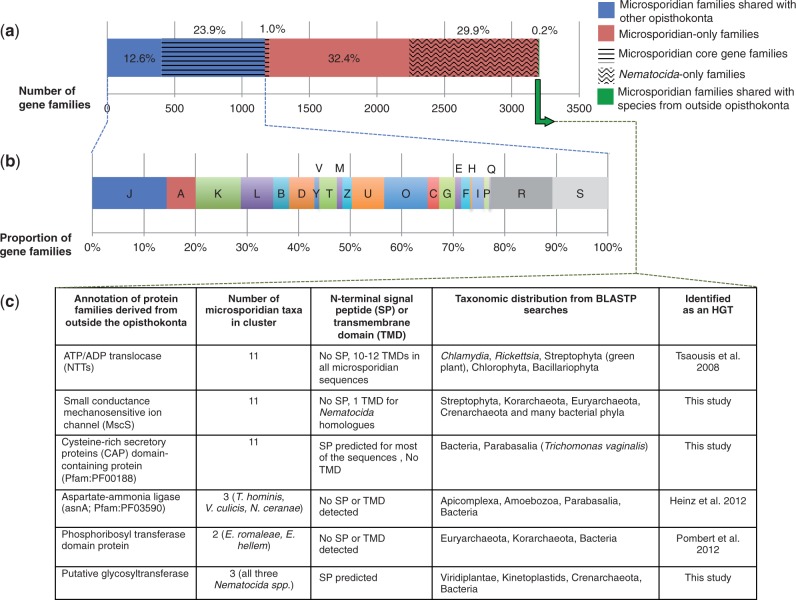
Origin of protein families in Microsporidia. (*a*) The proportion of microsporidian protein families that have homologs in other opisthokonta (blue), are microsporidian specific (red), or only have homologs in taxa outside the opisthokonta (green). Families conserved across at least nine microsporidian genomes were classified as core microsporidian gene families (striped background), whereas families that are only found in *Nematocida* isolates are highlighted with a wavy background. Notably, almost half of the identified microsporidian-only families are restricted to *Nematocida* spp., reflecting the large phylogenetic distance separating this lineage from the other Microsporidia (fig. 1). (*b*) Functional classification of microsporidian protein families shared with other opisthokonts (i.e., ancestral families). The retained families are distributed across all COG categories (Tatusov 2000). (*c*) Six microsporidian protein families potentially derived from horizontal gene transfer are listed. For each family, the number of Microsporidia that have homologs, the presence of predicted N-terminal SPs or TMDs, and the taxonomic distribution of the family are shown. J, translation, ribosomal structure, and biogenesis; A, RNA processing and modification; K, transcription; L, replication, recombination, and repair; B, chromatin structure and dynamics; D, cell cycle control, cell division, chromosomal partitioning; Y, nuclear structure; V, defense mechanisms; T, signal transduction; M, cell wall/membrane/envelope biosynthesis; N, cell motility; Z, cytoskeleton; U, intracellular trafficking, secretion, vesicular transport; O, posttranslational modification, protein turnover, chaperones; C, energy production and conversion; transport and metabolism of: G, carbohydrate; E, amino acids; F, nucleotides; H, coenzymes; I, lipids; P, inorganic ions; Q, secondary metabolites; R, general function prediction; S, unknown function.

We identified 3,204 protein families containing at least one sequence from the microsporidian proteomes sampled. Approximately 37% (1,171) of these protein families have homologs in other opisthokonts, whereas the remaining 63% are specific to one or more microsporidian genomes, in that no homologs for these families were detected outside the Microsporidia (using either BlastP (Altschul et al. 1990) or the more sensitive HHsearch (Söding 2005) (fig. 2). Despite extensive reductive evolution involving the loss of many protein families otherwise widely conserved among eukaryotes, microsporidian genomes have been accumulating these novel protein families throughout their evolution (fig. 1), raising questions about the functions they might perform. The uncharacterized families are unlikely to be sequencing artifacts or false-positive ORF calls because there is evidence of expression for 76% of them in at least one published proteomic or transcriptomic analysis (supplementary table S2, Supplementary Material online). These include proteomics of purified *T. hominis* or *E. cuniculi* spores (Brosson et al. 2006; Heinz et al. 2012) and RNA-Seq analyses of *Caenorhabditis elegans* cells infected with *N. parisii* (Cuomo et al. 2012) or of a rabbit cell line infected with *E. cuniculi* (Grisdale et al. 2013). Thus, differences in lineage-specific gene family content may reflect adaptation to different host cell environments.

In model eukaryotes, N-terminal SPs direct newly synthesized proteins to the secretory pathway, where proteins can be secreted from the cell, targeted to the plasma membrane or retained within the interconnected endomembranes of the secretory and endocytic pathways (rough endoplasmic reticulum, Golgi, endosomes, or lysosomes) (Ng 1996). Interestingly, approximately 72% of all microsporidian proteins with inferred SP are members of microsporidian-specific protein families (table 1), leading to a significant overrepresentation of proteins with SP in these families compared with those that microsporidians share with other eukaryotes (Fisher's exact test, *P* = 1.93 × 10^−^^171^). This suggests that proteins in novel microsporidian families might be secreted or targeted to the parasite cell surface or spore coat and hence could potentially be involved in host–parasite interactions. To test whether the acquisition of proteins with SPs occurred in the LCMA or in parallel during the radiation of Microsporidia, we repeated the test considering only the core microsporidian families (*P* = 6.05 × 10^−^^9^) or the complete data set without the core (*P* = 7.55 × 10^−^^172^). The enrichment of SPs in both subsets suggests that aspects of host interaction had already evolved in the LCMA but were further elaborated independently in different microsporidian lineages.

**Table 1:** Number of Homologous Sequences from Identified Protein Families in Analyzed Microsporidian Genomes

Microsporidian Species	Number of Predicted Protein-Coding Genes	Number of Protein Sequences in Protein Families		Number of Protein Sequences that Have Predicted N-Terminal SP in Protein Families	
		Ancestor Derived^a^	Microsporidian Specific^b^	Ancestor Derived^a^	Microsporidian Specific^b^
*Encephalitozoon cuniculi* GB-M1	1,996	1,349	636	32	70
*Encephalitozoon intestinalis* ATCC 50506	1,833	1,353	474	40	53
*Encephalitozoon hellem* ATCC 50504	1,848	1,365	478	33	56
*Encephalitozoon romaleae* SJ-2008	1,831	1,360	469	24	43
*Nosema ceranae* BRL01	2,060	1,264	503	40	69
*Vittaforma corneae* ATCC 50505	2,248	1,454	436	42	43
*Trachipleistophora hominis*	3,266	1,588	1,104	42	112
*Vavraia culicis floridensis*	2,780	1,478	929	52	139
*Nematocida sp1* ERTm2	2,770	1,287	1,256	30	160
*Nematocida parisii* ERTm1	2,661	1,234	1,406	36	162
*Nematocida parisii* ERTm3	2,726	1,251	1,432	36	165

^a^Ancestor derived: protein families that have homologs in other opisthokonts.

^b^Microsporidian specific: protein families that have no homologs outside the Microsporidia detected using either BlastP or HHsearch.

Microsporidian-specific protein families that are conserved across all microsporidian genomes are likely to be particularly important for maintenance of their parasitic lifestyle. Because genome data for most Microsporidia is incomplete, we defined “core” microsporidian-specific families as those present in at least 9 of the 11 genomes in this study. Thirty-two of these core families are specific to Microsporidia (i.e., they are found in at least 9 Microsporidia but not in any other sequenced genomes), of which only two—containing polar tube (Delbac et al. 2001; Peuvel et al. 2002) and spore wall (Xu et al. 2005; Brosson et al. 2006; Peuvel-Fanget et al. 2006; Southern et al. 2007) proteins—have at least one functionally characterized member (table 2 and supplementary table S3, Supplementary Material online). Both these families play an important role in the microsporidian lifecycle: polar tube proteins (PTPs) form part of the polar tube that Microsporidia use to penetrate the host cell upon germination (Franzen 2008), whereas the spore wall proteins form part of the resistant spores that Microsporidia use to survive outside the host cell (Brosson et al. 2006). Although the remaining 30 families are not yet characterized, members from all these families are transcribed during the infective stages of both *E. cuniculi* in infected rabbit kidney cells (Grisdale et al. 2013) and *N. parisii* in its nematode host (Cuomo et al. 2012) (supplementary table S3, Supplementary Material online), consistent with the hypothesis that they may play a broadly conserved role during infection. Four families (PTP2, Spore wall protein, and two uncharacterized families) also have at least one protein member supported by proteomics of purified *T. hominis* (Heinz et al. 2012) or *E. cuniculi* spores (Brosson et al. 2006; Grisdale et al. 2013) (table 2 and supplementary table S3, Supplementary Material online). Although their functions are largely unknown, four of these microsporidian-specific core families have multiple alpha-helical TMDs, suggesting they may be membrane proteins such as transporters (table 2). Even in the absence of such features, their conservation across the Microsporidia makes these families prime candidates for future experimental characterization to understand their functions in microsporidian biology.

**Table 2:** List of Microsporidian-Specific Core Protein Families

Protein Family Description	Predicted N-Terminal Signal Peptide (SP)	Predicted Transmembrane Domain (TMD)	Evidence for Expression			Family Name
			Trachipleistophora hominis	Encephalitozoon cuniculi	Nematocida parisii	
Polar tube protein 2	Yes	No	PS	PS, TIC	TIC	c_2006
Spore wall protein	Yes	No	PS	PS, TIC	TIC	c_1837
Hypothetical	No	4 TMDs	PS	TIC	TIC	c_1844
Hypothetical	No	4 TMDs	NA	TIC	TIC	c_2259
Hypothetical	No	1 TMD at the C-terminal region	—	TIC	TIC	c_1850
Hypothetical	No	2–3 TMDs	—	TIC	TIC	c_2033
Hypothetical	No	No	—	TIC	TIC	c_1437
Hypothetical	No	No	—	TIC	TIC	c_1513
Hypothetical	No	No	—	TIC	TIC	c_1594
Hypothetical	No	No	—	TIC	TIC	c_1703
Hypothetical	No	No	—	TIC	TIC	c_1704
Hypothetical	No	No	—	TIC	TIC	c_1710
Hypothetical	No	No	—	TIC	TIC	c_1718
Hypothetical	No	1–2 TMDs in 4 of 11 sequences	—	TIC	TIC	c_1838
Hypothetical	No	No	—	TIC	TIC	c_1845
Hypothetical	SP detected in 6 of 13 sequences	No	—	TIC	TIC	c_1846
Hypothetical	No	No	—	TIC	TIC	c_1853
Hypothetical	No	No	—	TIC	TIC	c_1855
Hypothetical	No	No	—	TIC	TIC	c_1856
Hypothetical	No	1 TMD in 2 of 11 sequences *T. hominis*, *V. culicis*)	—	TIC	TIC	c_1859
Hypothetical	No	No	—	TIC	TIC	c_1864
Hypothetical	No	No	—	TIC	TIC	c_2013
Hypothetical	No	No	—	TIC	TIC	c_2021
Hypothetical	No	No	—	TIC	TIC	c_2027
Hypothetical	SP detected in 3 of 12 sequences	No	—	TIC	TIC	c_2028
Hypothetical	No	No	—	TIC	TIC	c_2035
Hypothetical	No	No	—	PS, TIC	TIC	c_2038
Hypothetical	No	No	—	TIC	TIC	c_2261
Hypothetical	No	No	—	TIC	TIC	c_2270
Hypothetical	No	No	—	TIC	TIC	c_893
Hypothetical	No	1 TMD at the C-terminal three *Nematocida* sequences	—	TIC	TIC	c_2268
Hypothetical	No	No	NA	TIC	TIC	c_2004

Note.—PS, proteomics of purified spores; TIC, transcriptomics of cells infected with Microsporidia; NA, no homologs detected in the respective genome.

### The Microsporidian Core Gene Set Retains Essential, Highly Connected, and Highly Expressed Ancestral Genes

**Fig. 3.—:**
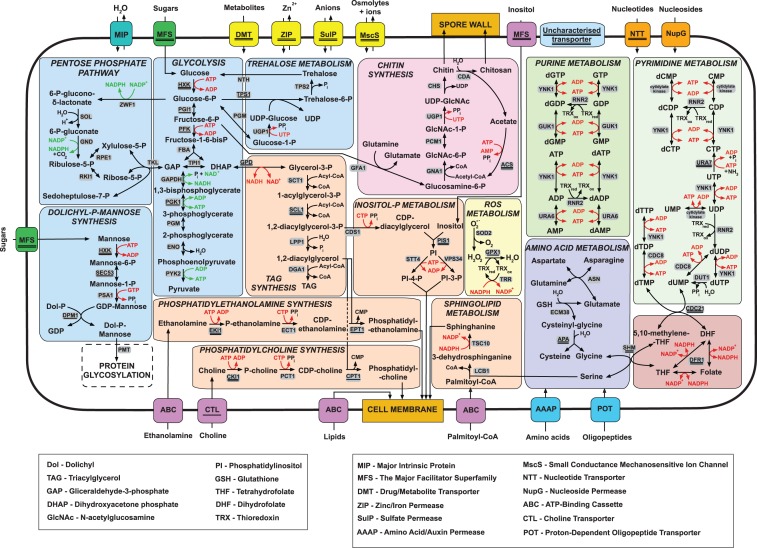
A model for the core metabolism and transporter repertoire of microsporidians. Metabolic enzymes and transporters that are conserved across at least 9 (out of 11) microsporidian genomes analyzed in this study are considered as microsporidian core genes. Arrows indicate metabolic enzymes that were identified in the microsporidian core gene set. Yeast gene name of enzymes catalyzing each reaction are highlighted on a gray background. Underlined gene names indicate genes with duplicated copies identified in one microsporidian genome, whereas double underlines denote genes that have undergone duplication in at least two microsporidian genomes.

Ninety-five percent (767/802) of the core microsporidian families have homologs in other opisthokonts and are likely to have been inherited vertically from their opisthokont common ancestor. In addition to genes involved in basic cellular functions such as transcription, translation, DNA replication and repair, cell cycle control, protein folding/turnover, intracellular trafficking, and mitochondrial and cytosolic Fe-S cluster assembly (fig. 2*b* and supplementary table S4, Supplementary Material online), our analysis shows that the microsporidian core gene set includes genes encoding all the key enzymes for glycolysis, the pentose phosphate pathway, trehalose metabolism, and chitin biosynthesis (fig. 3). Other enzymes broadly conserved across the Microsporidia are involved in the biosynthesis of known structural components of the fungal cell membrane and spore wall, including chitins, chitosans, and some major phospholipids found in the cell membranes of *S. cerevisiae* and other model eukaryotes, such as phosphatidylinositol, phosphatidylethanolamine, and phosphatidylcholine (Carman and Han 2011) (fig. 3). Overall, our comparative analyses reveal how the intracellular parasitic lifestyle of Microsporidia has shaped their highly reduced metabolism (fig. 3): Rather than investing in the energetically expensive de novo biosynthesis of basic biological building blocks (e.g., amino acids, sugars, nucleotides, and lipids) and cofactors (e.g., ATP, NAD+, and NADP+), they may instead make use of an expanded repertoire of cell surface transporters (see fig. 3 and below) to steal these essential molecules from the host.

The presence in the microsporidian core gene set of protein mannosyltransferase (see fig. 3), the key enzyme for O-mannosylation, and the evidence for mannosylation of *E. cuniculi* PTPs and spore wall proteins (Xu et al. 2005; Bouzahzah and Weiss 2010) suggest that mannosylated proteins play an important role in the parasitic lifestyle of Microsporidia, possibly as virulence factors as has been shown for several fungal pathogens (Fernández-Álvarez et al. 2009; Willger et al. 2009). Our analysis also reveals that the reported loss of genes involved in core carbohydrate and fatty acid metabolism in *Ent. bieneusi* (Akiyoshi et al. 2009) has occurred relatively recently in evolution, as we detected most of these genes in the most closely related genome available, that of *Vi. corneae* (Mittleider et al. 2002) (fig. 1). The genome of *Vi. corneae* encodes the key enzymes for glycolysis, the pentose phosphate pathway, and trehalose metabolism as well as fatty acid biosynthesis.

The widespread loss in Microsporidia of gene families otherwise conserved across eukaryotes raises the question of why some genes and pathways, but not others, have been retained across the majority of microsporidian lineages; presumably, the retained gene families play particularly important roles in the physiology of the parasite. To address this question, we investigated the functional properties of *S. cerevisiae* orthologs of the core microsporidian gene set; we chose *S. cerevisiae* for this analysis because it is the best studied relative of Microsporidia for which extensive functional data are available (Cherry et al. 2012). We observed that yeast genes that have an ortholog in the microsporidian core set are significantly more likely to be essential than those whose that do not (Fisher’s exact test, *P* = 2.47 × 10^−^^73^). Further, these yeast genes also have a significantly greater number of interaction partners (Wilcoxon rank-sum test, *P* = 2.5 × 10^−^^25^) and are expressed at significantly higher levels under normal growth conditions in yeast (Ghaemmaghami et al. 2003) (Wilcoxon rank-sum test, *P* = 2.5 × 10^−^^40^). All these measures suggest that the genes retained in the microsporidian core set are of greater functional importance than those that have been lost. However, these three properties are all correlated with each other: essential genes tend to be involved in more interactions than nonessential genes (Wilcoxon rank-sum test, *P* = 1.29 × 10^−^^30^ for our data set) and tend to be expressed at higher levels (*P* = 1.8 × 10^−^^15^), whereas the expression levels of yeast proteins correlate with numbers of interactions (*P* = 0.002, Spearman’s rank correlation). Thus, the possibility remained that only one of these properties was sufficient to explain the retention of these genes in the microsporidian core set. To evaluate this possibility, we fit a generalized linear model in which essentiality, expression level, and number of protein interaction partners were included as explanatory variables. All three terms were significant (essentiality: 0.97, *P* = 2.9 × 10^−^^32^; expression level: 8.2 × 10^−^^6^, *P* = 1 × 10^−^^7^; number of interactions: 0.02, *P* = 4.6 × 10^−^^14^), suggesting that all three factors contribute to retention in the microsporidian core set. Thus, as has been observed both in bacterial endosymbionts (McCutcheon and Moran 2011) and intracellular pathogens (Williams and Fares 2010), essential, highly expressed, and highly connected genes are preferentially retained during the reductive evolution of Microsporidia, presumably because of their greater functional importance to the cell. To evaluate whether this increased importance is reflected in the available functional data for Microsporidia, we compared the expression levels of *E. cuniculi* genes in the core set to the rest of the *E. cuniculi* genome using data from a recently published transcriptome analysis (Grisdale et al. 2013). The expression of core genes was significantly higher than that of other genes across all three experimental time points (±0.239 for core/noncore genes, 95% Bayesian credible interval [0.1818, 0.2975] for the effect of core genes), and we observed the same effect in the available *Nematocida* transcriptomic data (Cuomo et al. 2012) (±0.7909 for core/noncore genes, 95% Bayesian credible interval [0.716, 0.865] for the effect of core genes). These results suggest that the association between functional importance and high levels of expression is conserved in Microsporidia. We also found that the microsporidian core gene set is enriched for genes of archaeal origin (Fisher’s exact test, *P* = 5.24 × 10^−^^23^), consistent with the preferential retention of these genes in other reduced eukaryotic genomes (Alvarez-Ponce et al. 2013). In model eukaryotes, genes of bacterial ancestry outnumber those of archaeal ancestry (Esser et al. 2004; Cotton and McInerney 2010), but the archaeal genes are of greater functional importance (Cotton and McInerney 2010). These results can be understood in terms of the complexity hypothesis (Jain et al. 1999; Cohen et al. 2011), which posits that genes participating in a large number of interactions are less vulnerable to replacement by horizontal transfer because of the increased probability of disruption to the interaction network. Current hypotheses for eukaryotic origins suggest that the host cell for the mitochondrial endosymbiont was either a fully fledged Archaeon or a relative of the Archaea (Embley and Martin 2006). Thus, the relatively small number of archaeal genes that survive on modern eukaryotic genomes may represent the portion of this ancestral genome that was most resistant to replacement by bacterial HGT. Our finding that these highly connected archaeal genes are also preferentially retained during reductive evolution draws an interesting parallel between the survival of gene lineages in the face of replacement by HGT on the one hand and during genome reduction on the other.

### Expansion of a MscS Containing Protein Family in the Ancestral Microsporidian: Duplications and Horizontal Gene Transfer

**Fig. 4.—:**
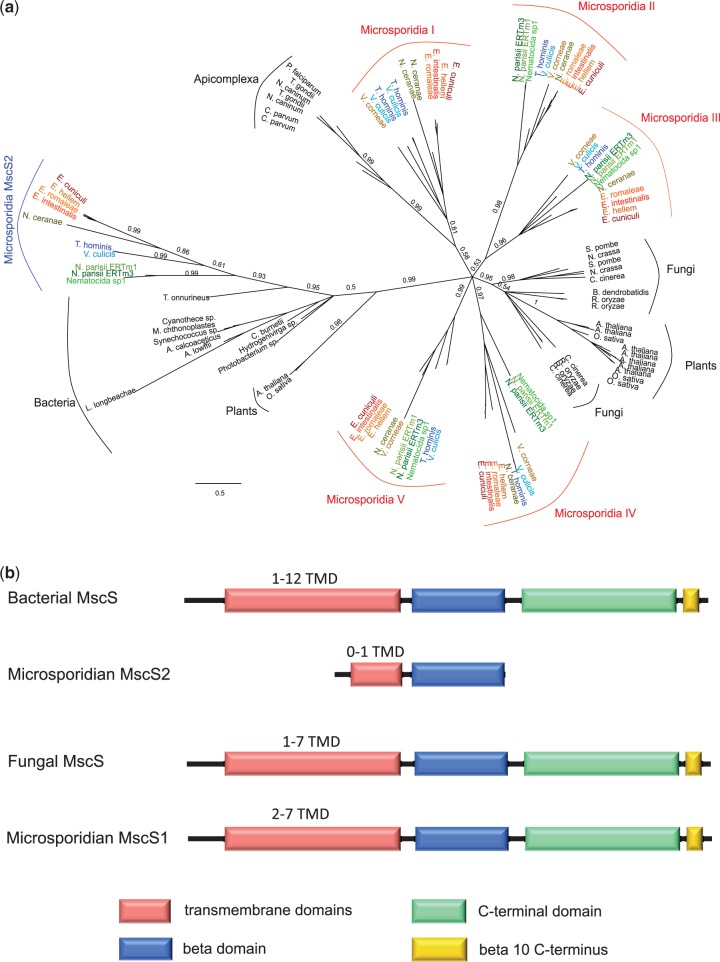
Duplication and horizontal transfer of a small MscS-domain-containing protein family. (*a*) Phylogeny of MscS homologs from Microsporidia, other eukaryotes, and bacteria. Microsporidian MscS homologs can be divided into two distantly related clusters: MscS I–V, which are related to the MscS genes of other eukaryotes, and MscS2, which cluster with bacterial MscS, consistent with a horizontal transfer from bacteria into the common ancestor of the Microsporidia; MscS I–V are derived from duplication of the eukaryotic-type MscS in the LCMA. The tree was inferred using the CAT60 model in PhyloBayes; branch lengths are proportional to the number of substitutions per site, as indicated by the scale bar. (*b*) Comparison of domain architectures among MscS homologs from Microsporidia, other eukaryotes, and bacteria. The canonical eukaryotic MscS homologs in Microsporidia have a conserved structural organization, whereas the bacteria-like MscS have undergone drastic reduction. The domain architecture of the bacterial MscS is drawn based on the domains identified in the structural data for the *Escherichia coli* YggB protein (Bass et al. 2002).

Compared with other eukaryotes, Microsporidia encode an expanded repertoire of MscS-containing proteins, with at least five copies in each genome analyzed (fig. 4*a*). MscS proteins regulate osmotic homeostasis by opening or closing a channel permeable to water and small ions in response to mechanical deformation of the cell membrane, such as that caused by physical or osmotic pressure (Kung et al. 2010; Haswell et al. 2011); MscS also function in the septum ring formation of bacteria and plastids (Wilson and Haswell 2012). Phylogenetic analysis indicates that microsporidian MscS have two distinct origins: one subfamily is related to eukaryotic MscS proteins, whereas the other was acquired from bacteria by horizontal transfer (fig. 4*a*). The bacterial-like microsporidian MscS (MscS2) is present as a single copy in each microsporidian genome, and our phylogeny indicates a single acquisition event at the LCMA (fig. 4*a*). We also detected MscS2-like sequences in some plants (the *Arabidopsis thaliana* homolog is located in the chloroplast [Froehlich et al. 2003]), which appear to have a separate origin to the microsporidian genes (fig. 4*a*).

In contrast, at least four copies of the eukaryotic-like microsporidian MscS (MscS1) are found in each microsporidian genome (fig. 4*a*, supplementary table S5, Supplementary Material online). Phylogenetic analysis indicates the presence of five copies at the LCMA (fig. 4*a*), suggesting that repeated gene duplications occurred in the ancestral microsporidian and that one copy was later lost in the lineage leading to *Nematocida* spp. One of the *T. hominis* eukaryote-like MscS (locus tag: THOM_1684) was present in the spore proteomics analysis (Heinz et al. 2012), and all MscS genes identified in *N. parisii* ERTm1 and *E. cuniculi* were detected in the transcriptomic analyses of cells infected with these parasites (Cuomo et al. 2012; Grisdale et al. 2013). In *E. cuniculi,* the two distinct MscS families were expressed differently across the infectious cycle of the parasite. Four of five *E. cuniculi* eukaryotic-like MscS genes significantly increased in expression between 24 h and 48 h postinfection (ECU01_1240: *P* = 0.01; ECU01_1170: *P* = 1.17 × 10^−^^3^; ECU10_1360: *P* = 1.14 × 10^−^^7^; ECU03_1000: *P* = 1.02 × 10^−^^6^). In contrast, the level of expression of bacterial-like MscS significantly decreased during the same time period (ECU09_0470: *P* = 1.02 × 10^−^^6^). The presence of two distinct MscS families in Microsporidia, one obtained by horizontal transfer and the other by lineage-specific expansion, coupled with their expression in both the spore and meront stages and throughout the parasite life cycle, suggest that MscSs play important, but so far unrecognized, roles in microsporidian biology. Based on what is known about the function of these proteins in model organisms, these roles may be related to the regulation of osmotic stress at the cell surface during different life stages (e.g., during the germination process), as well as during cell or organelle division (Wilson and Haswell 2012).

Comparing the domain architecture of the two microsporidian MscS families, the MscS1 family resembles a “canonical” MscS protein, whereas the bacterial-like MscS2 sequences are extremely reduced in length (fig. 4*b*). Compared with the structure of *Escherichia coli* MscS (Bass et al. 2002), the MscS2 sequences have lost the C-terminal domain and most, or all, of the TMDs, with only the beta-domain being retained (fig. 4*b*). In *Es. coli*, MscS is characterized by three TMDs (Bass et al. 2002), the C-terminal domain and the third TMD are important for channel function and gating (Koprowski and Kubalski 2003; Edwards et al. 2005). It is therefore unclear whether any of the microsporidian MscS2 proteins are membrane bound, and if so whether they function as ion channels.

### Horizontal Gene Transfers into Microsporidia

Horizontal gene transfer into Microsporidia appears to be relatively rare compared with other eukaryotic parasites such as *Entamoeba histolytica* and *Trichomonas vaginalis* (Alsmark et al. 2013), perhaps because of their obligate intracellular lifestyle. Nonetheless, the small numbers of HGTs that have been detected are important for microsporidian biology, such as the nucleotide transporter (NTT) proteins that Microsporidia may use to steal host ATP (Tsaousis et al. 2008; Selman and Corradi 2011; Heinz et al. 2012). Our analysis shows that homologs of most microsporidian genes are either broadly distributed among eukaryotes or specific to extant microsporidian lineages (fig. 2*a*). However, we did identify six families with a phyletic pattern suggesting that they may have been acquired by the Microsporidia through HGT from lineages outside the opisthokonta (fig. 2*c*). These candidate HGTs include some that were previously known, including the NTTs (Tsaousis et al. 2008), an aspartate-ammonia ligase (Heinz et al. 2012), and a phosphoribosyltransferase (Pombert et al. 2012). In addition to these families and the MscS described above, we identified two new candidate HGTs: a glycosyl transferase and a CAP-domain containing protein.

#### Glycosyl Transferase

Glycosyl transferase (GT; EC 2.4.x.x) catalyzes the transfer of a sugar moiety during the formation of a glycosidic bond during the biosynthesis of glycoproteins and glycolipid sugars (Campbell et al. 1997). One of the GT families is only present in the three *Nematocida* species (e.g., NEPG_01588). Proteins in this family contain a glycosyl transferase group 1 domain (GT1; Pfam:PF00534) and show a high level of protein sequence similarity (40–50%) to homologs from Viridiplantae, Kinetoplastids, and several bacteria but no BlastP hits (*e*-value cutoff <0.001) to sequences from other Microsporidia or fungi. Phylogenetic analysis strongly supports a HGT from bacteria to *Nematocida* spp. (supplementary fig. S1, Supplementary Material online). Although most nonmicrosporidian homologs do not have an N-terminal SP that could target proteins to the secretory pathway, two of the three *Nematocida* sequences do, suggesting acquisition of SP in the *Nematocida* GT1 family. One possibility is that these proteins are secreted into the host cytosol to interfere with host metabolism; a similar scenario was recently proposed for a family of *Nematocida* hexokinases with SPs that can be recognized by the *S. cerevisiae* secretory pathway (Cuomo et al. 2012).

#### CAP Protein Family

**Fig. 5.—:**
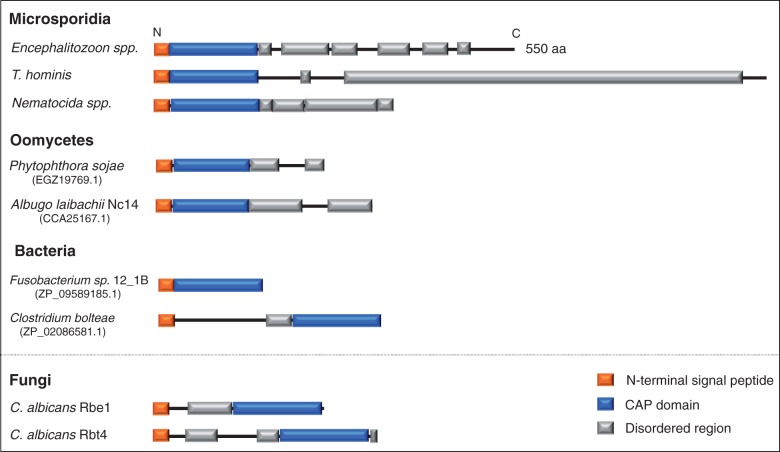
Comparison of domain architectures among cysteine-rich secretory protein (CAP) domain containing homologs. The N-terminal CAP domain is conserved across the microsporidian CAP-containing protein homologs. The microsporidian CAP-containing region shows significant similarity to sequences from bacteria, oomycetes, and *Dictyostelium* but not to sequences from other opisthokonta (supplementary table S6, Supplementary Material online). The sequence regions outside the CAP domain are not conserved among distantly related microsporidian species and are predicted to be intrinsically disordered, as observed for some CAP homologs from fungi and bacteria. Characterized CAP-containing proteins often possess N-terminal secretory signals and are secreted (Gibbs et al. 2008; Choudhary and Schneiter 2012; Röhm et al. 2013) from cells; because all microsporidian CAP-containing proteins possess SPs, the same may be true for these proteins. Domain architecture is drawn to scale—CAP domain region identified by InterproScan (Zdobnov and Apweiler 2001), disordered region inferred by MESSA (Cong and Grishin 2012), and N-terminal secretory signal detected by SignalP (Petersen et al. 2011).

CAP (which stands for Cysteine-rich secretory proteins, antigen S, and pathogenesis-related 1 proteins; Pfam:PF00188) domains are found in proteins with a broad range of functions, including immunity in animals, antifungal activity in plants (Gibbs et al. 2008; Choudhary and Schneiter 2012), and pathogenicity in fungi such as *Candida albicans* (Prados-Rosales et al. 2012; Röhm et al. 2013); many CAP domain-containing proteins are secreted (Gibbs et al. 2008; Choudhary and Schneiter 2012; Röhm et al. 2013). Microsporidia encode a protein family with an N-terminal CAP domain, in which the first 150 amino acids show significant similarity (BlastP *e* value <1 × 10^−^^5^) to sequences from bacteria, oomycetes, and *Dictyostelium* but not to sequences from other opisthokonta (supplementary table S6, Supplementary Material online). These positions are confined to the N-terminal CAP domain itself, with no detectable similarity outside this region except among closely related species (e.g., between *Encephalitozoon* spp.). Phylogenies of this gene family are poorly resolved because fewer than 100 amino acid positions could be reliably aligned among the microsporidian and related sequences. Nonetheless, the patchy taxonomic distribution of BlastP hit results (supplementary table S6, Supplementary Material online) and the broad conservation of this gene family among microsporidians (one copy per taxa) suggest that the microsporidian CAP-containing proteins, whatever their evolutionary origins, were present in the LCMA. Members of this family were detected both in proteomics analysis of purified *T. hominis* spores, in RNAseq data of *C. elegans* cells infected with *N. parisii* and of RK-13 cells infected with *E. cuniculi* (Cuomo et al. 2012; Heinz et al. 2012; Grisdale et al. 2013). In *E. cuniculi,* a significant increase in CAP gene expression (ECU03_0990 *P* = 9.41 × 10^−^^10^) was detected between 24 and 48 h postinfection, similar to the pattern of expression of genes involved in spore formation (e.g., spore wall and PTPs) (Grisdale et al. 2013). Similarly, the expression of *N. parisii* CAP gene (NEPG_01805) increased at the time point in which spores were first observed (Cuomo et al. 2012). Interestingly, the nonconserved regions are predicted to be intrinsically disordered using MESSA (Cong and Grishin 2012) (see Materials and Methods, fig. 5). Such regions cannot typically form stable tertiary structures in isolation and are often stabilized through interactions with other proteins (Dyson and Wright 2005), allowing them to bind to a wide range of substrates or interacting partners (Radivojac et al. 2007). Whatever their taxonomic origin, it appears likely that the microsporidian CAP-containing protein family may have an important role in interacting with host cells and possibly pathogenicity and is worth investigating experimentally.

### Expansion and Functional Divergence of Conserved Ancestral Families

The dominant mode of microsporidian genome evolution during the transition to intracellular parasitism appears to have been loss of genes that are otherwise broadly distributed among eukaryotes (fig. 1). As a consequence, microsporidian gene families that run counter to this trend are potentially interesting, as they might represent adaptations to the intracellular parasitic niche (Vogel and Chothia 2006). To systematically identify cases of microsporidian-specific expansions of vertically inherited gene families, we parsed the tree topologies of each gene family to identify microsporidian-specific expansions (see Materials and Methods). We obtained 225 trees containing microsporidian-specific duplications of conserved opisthokont genes (supplementary table S5, Supplementary Material online). The detected microsporidian-specific expansions affect all COG functional categories (Tatusov 2000) and many core microsporidian gene that are part of metabolic pathways (fig. 3, supplementary table S5, Supplementary Material online), including genes coding for enzymes (e.g., hexokinases and zinc metalloprotease Ste24), chaperones (e.g., Hsp90), and several transporter families.

#### Hexokinases

Phylogenetic analysis indicates that a family of microsporidian hexokinases has undergone lineage-specific gene family expansion twice independently: once in the lineage leading to *T. hominis* and *V. culicis* and also in that leading to *Vi. corneae* (supplementary fig. S2, Supplementary Material online). Independent duplications of this gene have also taken place in fungi and animals. Interestingly, although none of the animal or fungal homologs have predicted N-terminal SP, most of the microsporidian species analyzed possess at least one copy of hexokinase with a predicted N-terminal SP that potentially targets protein to the secretory pathway (supplementary fig. S2, Supplementary Material online). This is consistent with the hypothesis that some microsporidian hexokinases might be secreted into the host cytosol or targeted to specific cellular compartments (Cuomo et al. 2012).

The acquisition of SP in microsporidian hexokinases, an enzyme normally part of glycolysis in the cytosol, prompted us to search for any other gene families whose microsporidian members may have acquired SPs. After correction for multiple testing, our screen identified only two additional families with a significant (*Q* value < 0.05) overrepresentation of SP among microsporidian homologs when compared with fungal outgroups (supplementary table S7, Supplementary Material online). These include a family of LRR-containing proteins and mannosyltransferases (supplementary table S7, Supplementary Material online). The presence of SP on mannosyltransferases is not unexpected because they are localized to the Golgi in *S. cerevisiae* (Lussier et al. 1996). However, the enrichment of SP in LRR-containing proteins is interesting as repetitive proteins—including LRRs—are a recurring feature of the genomes of pathogenic bacteria, fungi, and protozoa (Fankhauser et al. 2007; Butler et al. 2009).

#### Metalloprotease Ste24

Homologs of the zinc metalloprotease Ste24 also appear to have undergone multiple independent expansions in Microsporidia. With the exception of *Nematocida* spp., all microsporidian genomes sampled encode at least two copies, with additional lineage-specific expansion in *Nosema* and *Encephalitozoon* (supplementary fig. S3, Supplementary Material online). One clade of microsporidian Ste24 genes is highly divergent from other eukaryotic Ste24 sequences (supplementary fig. S3, Supplementary Material online). The top Blast hits of sequences from this clade include bacterial sequences, but HGT from bacteria is not supported by phylogenetic analysis. Instead, the phylogeny is consistent with a duplication of Ste24 in the LCMA followed by high levels of sequence divergence in one copy (supplementary fig. S3, Supplementary Material online). All the microsporidian Ste24 homologs share common features with yeast Ste24, which has the zinc metalloprotease catalytic motif HEXXH and multiple TMDs (Tam et al. 2001), suggesting that they are membrane-spanning proteases. A potentially relevant function of yeast Ste24 is its role in the localization of chitin synthases 3 (Chs3) to the plasma membrane, particularly at the bud neck during cell division (Meissner et al. 2010). Chs3 is required for the synthesis of chitin (Meissner et al. 2010), the primary component of the inner layer of the microsporidian spore (the endospore). Interestingly, the expression of all three *E. cuniculi* Ste24 genes increased significantly (ECU02_1380: *P* = 2.29 × 10^−^^11^; ECU05_1370: *P* = 0.035; ECU05_1390: *P* = 1.99 × 10^−^^10^) as the infectious cycle progresses, similar to the expression pattern of genes coding for spore components (Grisdale et al. 2013). Further, expression of the single copy of Ste24 in *N. parisii* (NEPG_00127) also increased with the first observation of spores (Cuomo et al. 2012). These findings suggest that Ste24 may have retained this role in the regulation of chitin synthesis in Microsporidia, thus contributing to spore formation. The function of the highly divergent copy is unknown, but it is interesting to note that proteases in many pathogens have been identified as virulence factors targeting specific host proteins (Yike 2011).

#### Chaperone Hsp90

The chaperone repertoire of the Microsporidia has been reduced in variety and number in comparison to their fungal relatives, although all eight eukaryotic TriC/CCT paralogs have been retained (Heinz et al. 2012). An exception to this trend is the presence of two copies of the molecular chaperone Hsp90 in *T. hominis* and *V. culicis*. Based on the tree topology (supplementary fig. S4, Supplementary Material online), this second copy results from a gene duplication event before the radiation of the *T. hominis/V. culicis* and the *Nosema/Encephalitozoon* clades; the second duplicate appears to have been lost in *Encephalitozoon*, *Nosema,* and *Vittaforma*. The long branch leading to this second duplicate indicates that it is evolving more quickly than the copy conserved in all Microsporidia, suggesting functional divergence between the paralogs. Although the amino acid residues involved in ATP binding and the ATPase catalytic loop described for yeast Hsp90 (Pearl and Prodromou 2006) are conserved across all microsporidian Hsp90 sequences, the divergent copies are also predicted to possess N-terminal SPs, hinting at a possible role as a secreted protein. Hsp90 is a cell surface virulence factor in the pathogenic fungus *Can. albicans* (Burt et al. 2003), and overexpression of Hsp90 in *S. cerevisiae* increases virulence in mice (Hodgetts et al. 1996). Because of the large number of client proteins with which Hsp90 is known to interact (Zhao et al. 2005), it is difficult to speculate as to the role of this divergent duplicate without further functional characterization; nonetheless, the presence of additional copies in several microsporidians and their acquisition of secretory signals suggest this family may be worth exploring further.

#### Many Transport Protein Families Are Expanded within the Microsporidian Radiation

To compensate for their reduced biosynthetic capability, Microsporidia steal energy and nutrients from their host cell using transport proteins. The observed expansion of existing transporter families and gain of new genes (e.g., NTTs and MscS), following the initial loss of many plasma membrane transporters relative to yeast (Heinz et al. 2012), are probably to fill gaps in parasite metabolism, through neofunctionalization (i.e., the gain of a new function), subfunctionalization (e.g., expression during different life cycle stages), or gene dosage effects. In addition to the NTT and MscS families discussed earlier, eight other transport protein families also appear to have undergone duplication within the microsporidian radiation (supplementary tables S5 and S8, Supplementary Material online). These include putative major facilitator superfamily (MFS) sugar transporters (supplementary fig. S5, Supplementary Material online), H+/Na+ translocating ATPases, sulfate permeases (SulP) (supplementary fig. S6, Supplementary Material online), Zn2+/Fe2+ permeases (ZIP) (supplementary fig. S7, Supplementary Material online), choline transporter-like (supplementary fig. S8, Supplementary Material online), and drug/metabolite transporters (supplementary fig. S9, Supplementary Material online), as well as a family of putative transporters (≥4 TMDs) of unknown function (supplementary fig. S10, Supplementary Material online). Based on their similarity to protein sequences from model eukaryotes, seven of eight of these expanded transporter families are likely to be located on the parasite plasma membrane (supplementary table S8, Supplementary Material online). These results suggest that the expansion of these transport families was driven by the transition to an intracellular lifestyle where most of the nutrients can be acquired from the host cytosol. In several cases, the expansion of a transporter family by duplication was followed by reciprocal loss of functional domains in the resulting paralogs (for instance, the SulP and ZIP families, see supplementary figs. S6 and S7, Supplementary Material online, respectively), strongly suggesting functional divergence by subfunctionalization. In contrast, one microsporidian MFS paralog has gained a conserved insertion between the seventh and eighth TMDs (supplementary fig. S5, Supplementary Material online), suggesting that it may have undergone neofunctionalization. Some members of this subfamily are positive for the NupG nucleoside/H+ symporter profile (Pfam:PF03825) domain that is broadly distributed among bacteria but is found in only a limited number of eukaryotes (Microsporidia*, Trypanosomatidae*, *Dictyosteliida*, *Anopheles,* and *Drosophila*). A recombinant NupG in *Es. coli* transports both purine and pyrimidine nucleosides across the cell membrane (Xie et al. 2004). In our phylogenetic analysis (supplementary fig. S5, Supplementary Material online), putative microsporidian NupG formed a divergent clade that neither clustered with other eukaryotes nor with any bacterial group. Therefore, their evolutionary origins are currently unresolved. Nonetheless, it is tempting to speculate that this family of transporters might complement the NTTs (Tsaousis et al. 2008) in importing DNA and RNA precursors, particularly given the apparent absence of enzymes for de novo nucleoside biosynthesis in the Microsporidia (Heinz et al. 2012).

### Gene Order Conservation among Microsporidia

**Fig. 6.—:**
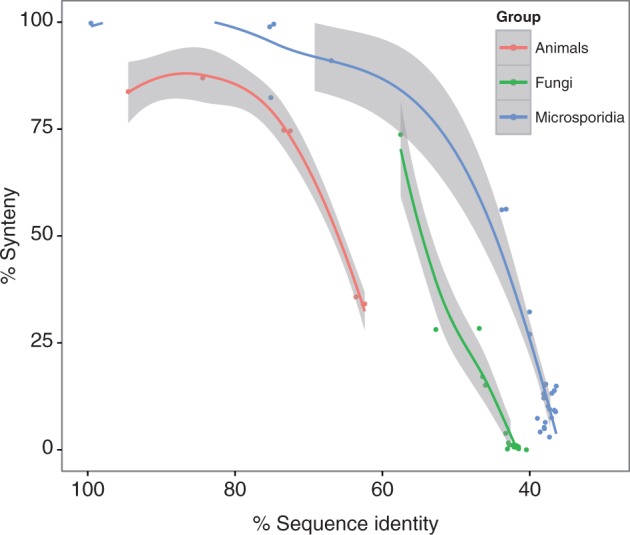
The decay of synteny with protein sequence divergence. The percentage of orthologs that are syntenic between all pairs of microsporidian genomes as a function of ortholog sequence identity (roughly, time). The trend in Microsporidia (blue) is compared with that in fungi (green) and animals (red). The relationships are visualized with Local Regression (LOESS) curves; the shading around these curves denotes 95% confidence intervals. Synteny decays with sequence identity in all three groups, but the relationship between the two is nonlinear. At a given level of sequence identity, synteny is higher between microsporidian genomes compared with fungi and animals. Modeling this relationship using generalized linear or additive models suggested that synteny decays more slowly in compact genomes, potentially explaining this observation (see text).

Previous studies (Slamovits et al. 2004; Corradi et al. 2007) based on comparisons of sequence data from *E. cuniculi*, *Antonospora Locustae,* and *Ent. bieneusi* have been interpreted to suggest that the conservation of synteny among microsporidian genomes is unexpectedly high compared with the rate of sequence evolution. Moreover, the conservation of pairwise synteny between *E. cuniculi* and *Ent. bieneusi* was similar to that between *E. cuniculi* and the more distantly related *A. locustae*, suggesting that the degree of gene order conservation might be relatively static across the group, regardless of the evolutionary distance between the lineages being compared (Corradi et al. 2007). This conservation of synteny would be especially striking if it extended to the larger microsporidian genomes that have recently become available, because these genomes differ markedly in other aspects of genome architecture such as overall size, length of intergenic regions and repetitive content. We therefore assessed the conservation of gene order in our expanded sample of 11 microsporidian genomes to evaluate the relationship between evolutionary divergence and conservation of synteny across the group. To quantify the level of synteny between pairs of microsporidian genomes, we calculated the proportion of orthologous pairs that are located next to each other in both genomes. To avoid underestimating the level of synteny, genes located at contig ends were excluded from our calculations. To compare gene order conservation among microsporidians to that in other opisthokonts, the same procedure was repeated for a selection of fungal and animal genomes. Our analyses confirm the previously reported finding that, relative to evolutionary divergence, the level of syntenic conservation is high throughout the Microsporidia (Slamovits et al. 2004; Corradi et al. 2007): at a given level of evolutionary divergence (protein sequence identity), microsporidian genomes show a greater level of gene order conservation than do animal or fungal genomes (fig. 6). However, our analysis does not support the notion of an invariant syntenic core for Microsporidia (Slamovits et al. 2004)—as with other eukaryotes, synteny decays with increasing evolutionary distance, presumably due to the accumulation of independent gene order rearrangements over time. This trend is now more apparent because of the improved sampling of genomes currently available; closely related Microsporidia such as the available *Nematocida* strains are highly syntenic (∼99.7%), but this value drops to below 20% among the sampled species that are more distantly related to each other. Contemporary Microsporidia have highly variable genome architecture (Williams et al. 2008; Heinz et al. 2012), which may influence the rate at which genomic rearrangements occur. For example, gene-sparse genomes (such as that of *T. hominis*) might better tolerate rearrangements because recombination or insertion events are less likely to disrupt an existing coding sequence than in gene-dense genomes such as those of the *Encephalitozoon* species. To test this hypothesis, we fit a generalized linear model to the microsporidian synteny data, modeling the proportion of syntenic pairs between species as a function of both protein sequence identity and the mean proportion of intergenic DNA in the two genomes being compared (see Materials and Methods). Both terms were significant (coefficient for sequence identity: 1.72, *P* < 10^−^^10^; coefficient for intergenic content: −33.46, *P* = 0.023), suggesting not only that synteny decreases with time but also that the decay of synteny occurs more rapidly in gene-sparse genomes. This result provides quantitative support for the influence of genome compaction in the conservation of synteny in the Microsporidia as a whole (Slamovits et al. 2004).

## Conclusions

The small (2.3–24 Mb) genomes of Microsporidia have made them an attractive model system for studying reductive evolution in eukaryotes. Until recently, however, the relative paucity of genome sequences has made it difficult to draw general conclusions about the evolution of the group. Our analysis of a broad sample of microsporidian genomes demonstrates that the tempo and mode of genome evolution in this group has been far more dynamic than previously appreciated: Following a massive loss of vertically inherited gene families in the LCMA, a process of gene family formation and expansion has substantially enlarged the core microsporidian proteome. This expansion of endogenous gene families has quantitatively outweighed other processes of gene gain such as horizontal transfer, although some particularly interesting families—such as the MscS-containing proteins—have experienced both modes of gene family expansion. Our analyses also provide evidence for functional divergence of some expanded microsporidian families—particularly transporters, where we observed reciprocal losses of functional domains indicating subfunctionalization (e.g., ZIP and SulP transporter families) and, in one case, gain of an additional domain, suggesting acquisition of a new function (the NupG family of putative nucleoside transporters). Most of these expanded transporter families are predicted to localize to the parasite cell membrane, suggesting that their function is to supply the minimalist microsporidian metabolism with metabolic precursors that would be energetically expensive to synthesize de novo. Rather than biosynthesize these fundamental biological building blocks, Microsporidia may use an expanded transporter repertoire to steal them from their hosts.

Our analyses reveal that the striking similarities between the Microsporidia and intracellular bacteria—such as small genome size, low coding capacity, and high AT-content—extend to the process of reductive evolution itself: Microsporidia retain essential, highly connected, and highly expressed ancestral genes while greatly reducing their metabolic capability, potentially evolving by a ratchet process similar to that which has been described for intracellular bacteria (McCutcheon and Moran 2011). Given these parallels, the recent identification of mutualistic or symbiotic Microsporidia (Haine et al. 2007) as observed for intracellular bacteria such as *Wolbachia* (Teixeira et al. 2008) is not entirely unexpected.

All microsporidian genomes sequenced to date encode large numbers of hypothetical, or uncharacterized, proteins. We have shown that these protein families are enriched for N-terminal secretory signals, motivating the hypothesis that many of them may be involved in host–parasite interactions. Uncharacterized protein families conserved across the Microsporidia are particularly likely to play important roles in their parasitic lifecycle, and there is evidence of expression of all these families in published proteomic or transcriptomic experiments; we suggest that these families represent particularly promising candidates for future experimental dissection of the microsporidian biology. Informed by the comparative analyses reported here, such approaches promise to further deepen our understanding of Microsporidia and their impact on host cells.

## Supplementary Material

Supplementary data, figures S1–S10, and tables S1–S8 are available at *Genome Biology and Evolution* online (http://www.gbe.oxfordjournals.org/).

Supplementary Data
