# Supplementary material for: Reduction and Expansion in Microsporidian Genome Evolution: New Insights from Comparative Genomics
Source: Genome Biol Evol. 2013 Nov 19;5(12):2285–303. doi: 10.1093/gbe/evt184 (PMC3879972; doi:10.1093/gbe/evt184)
Supplement: Supplementary Data [file supp_5_12_2285__index.html]

Reduction and expansion in microsporidian genome evolution: new insights from comparative genomics — Reduction and Expansion in Microsporidian Genome Evolution: New Insights from Comparative Genomics — Supplementary Data 

# Reduction and Expansion in Microsporidian Genome Evolution: New Insights from Comparative Genomics

## Supplementary Data

files

**Files in this Data Supplement:**

- Supplementary Data - pdf file
- Supplementary Data - doc file
- Supplementary Data - xlsx file
