# Supplementary figures and images for: Reduction and Expansion in Microsporidian Genome Evolution: New Insights from Comparative Genomics
Source: Genome Biol Evol. 2013 Nov 19;5(12):2285–303. doi: 10.1093/gbe/evt184 (PMC3879972; doi:10.1093/gbe/evt184)

Figure S1

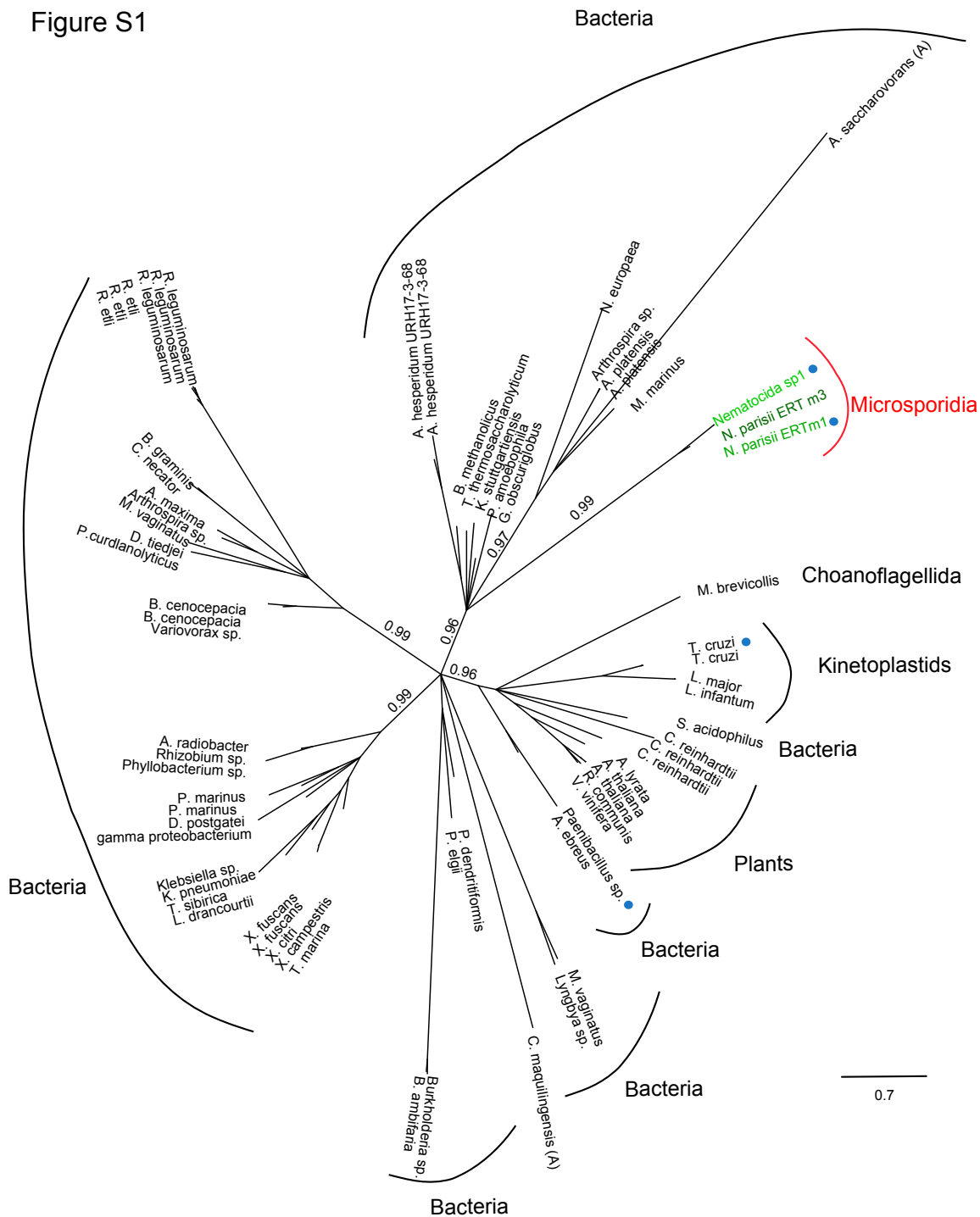

Figure S2

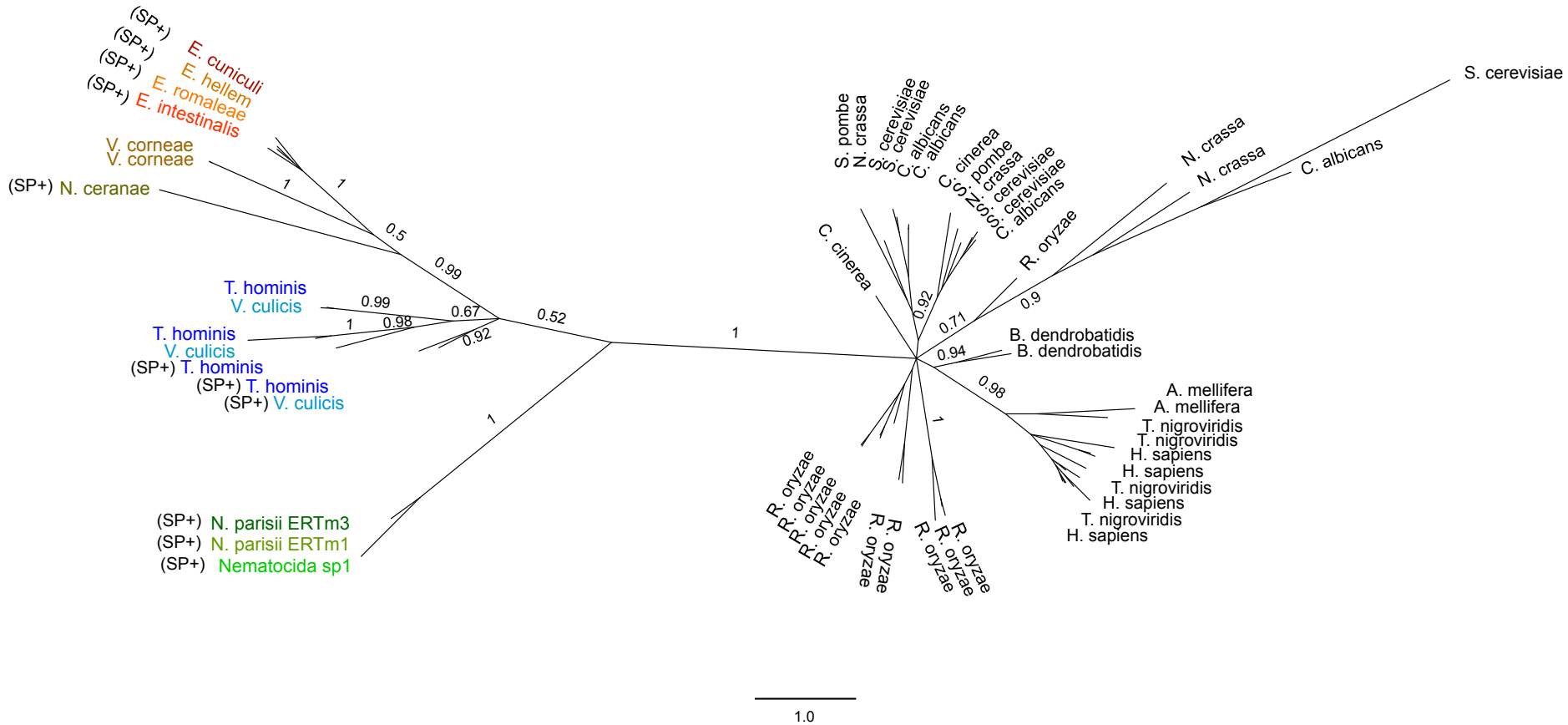

Figure S3

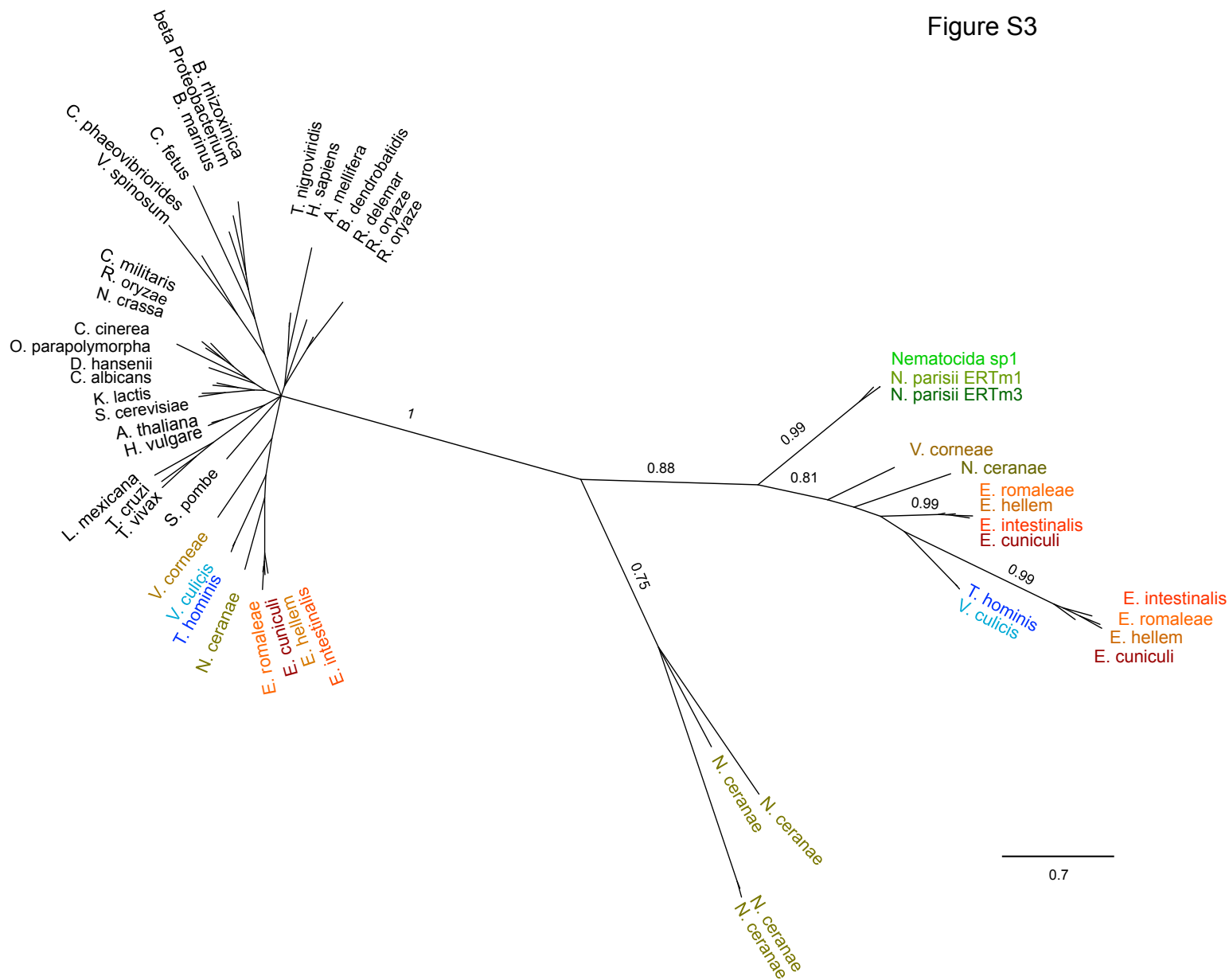

Figure S4

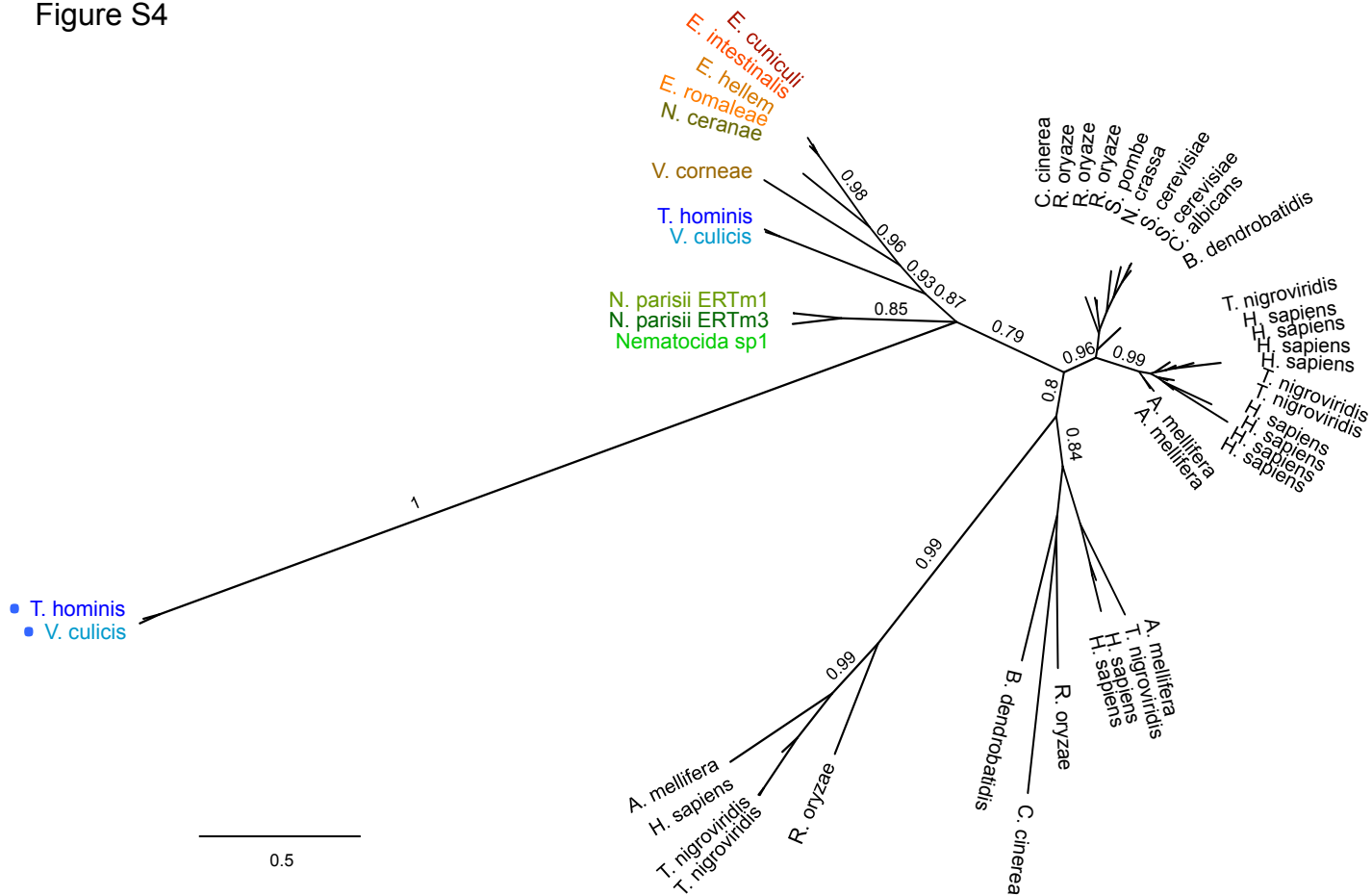

(a)

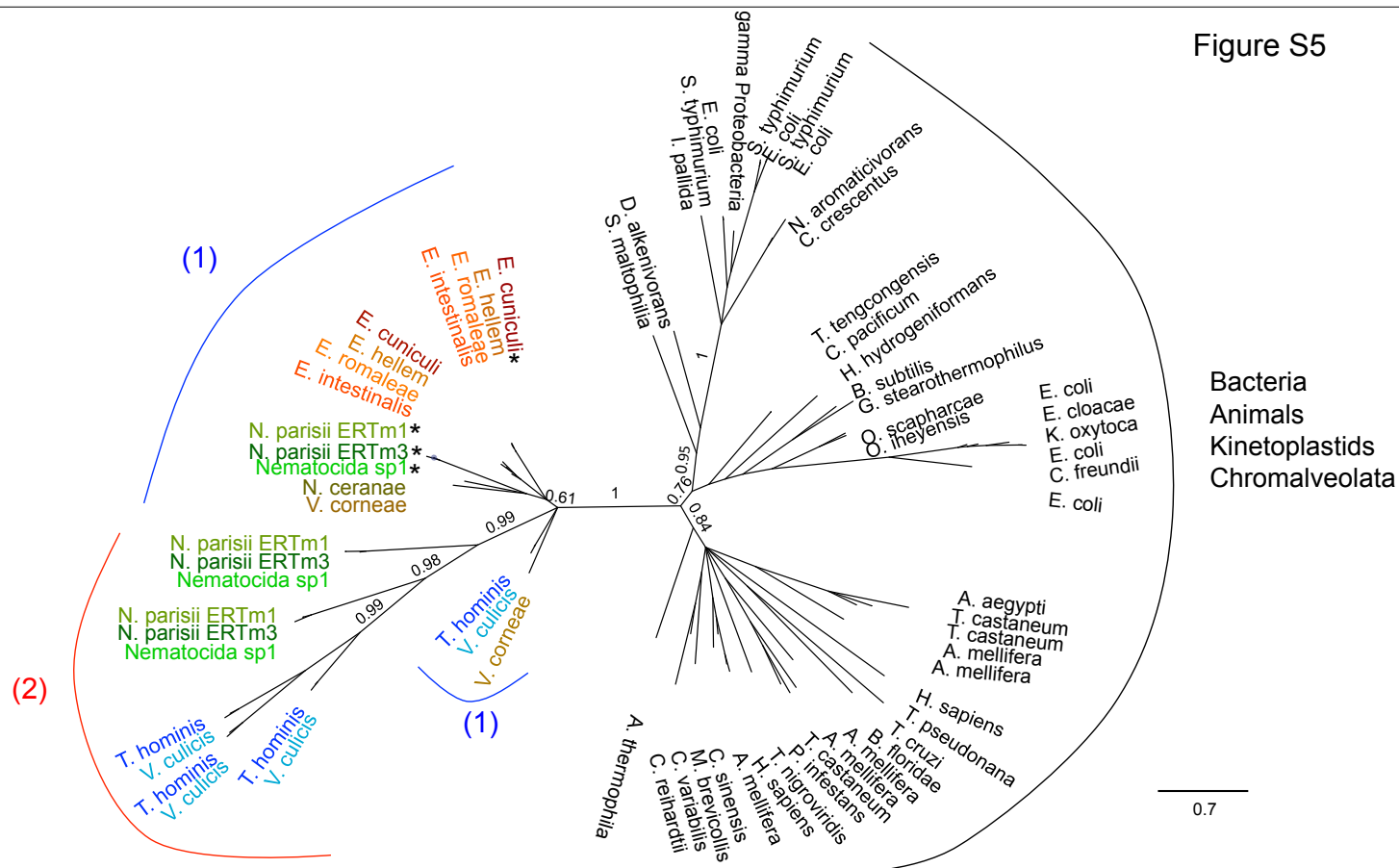

(b)

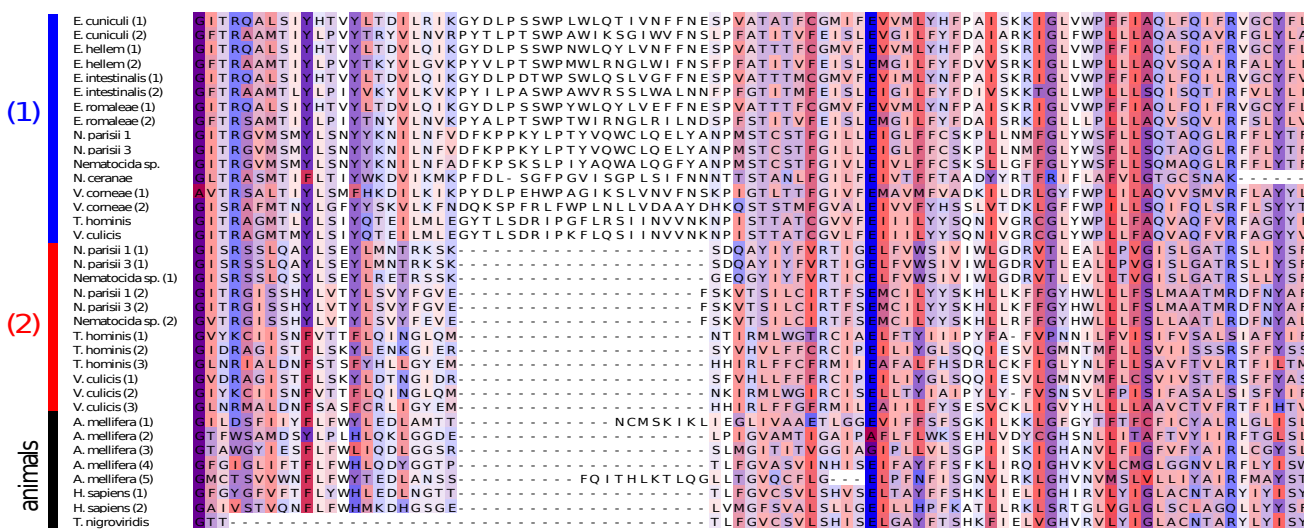

Figure S6

(a)

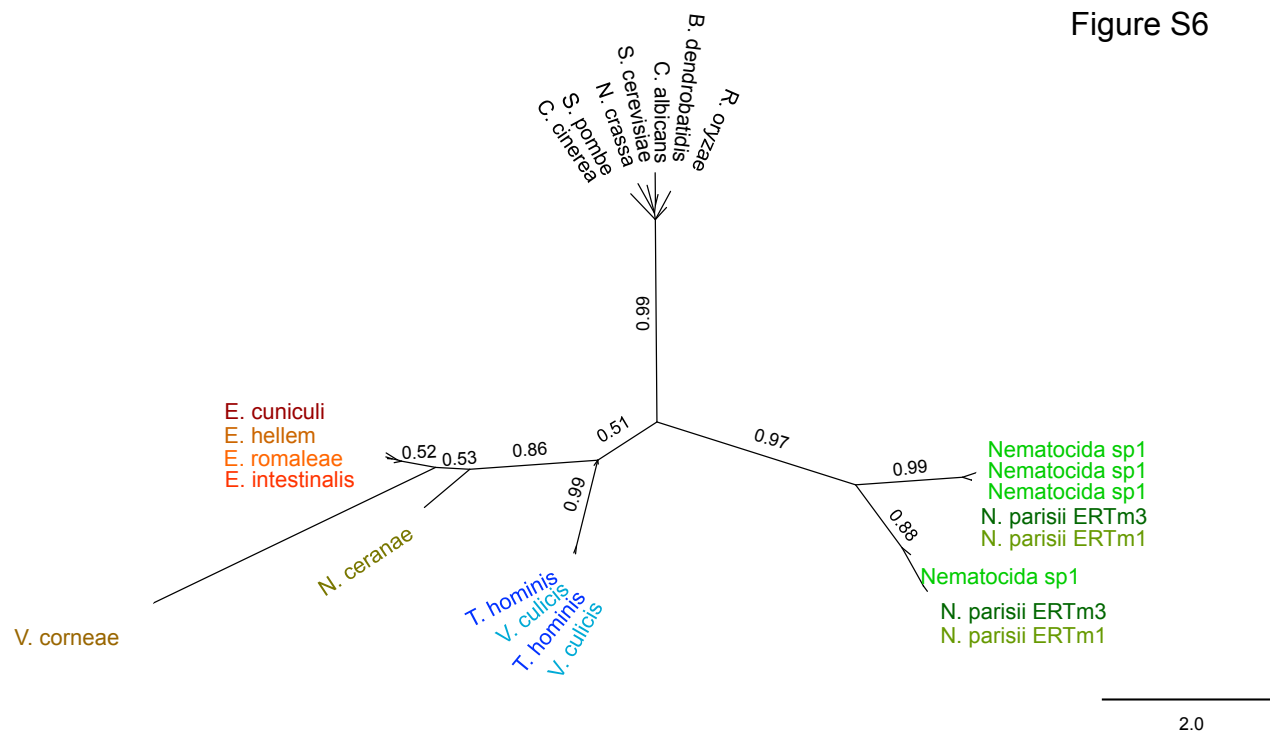

(b)

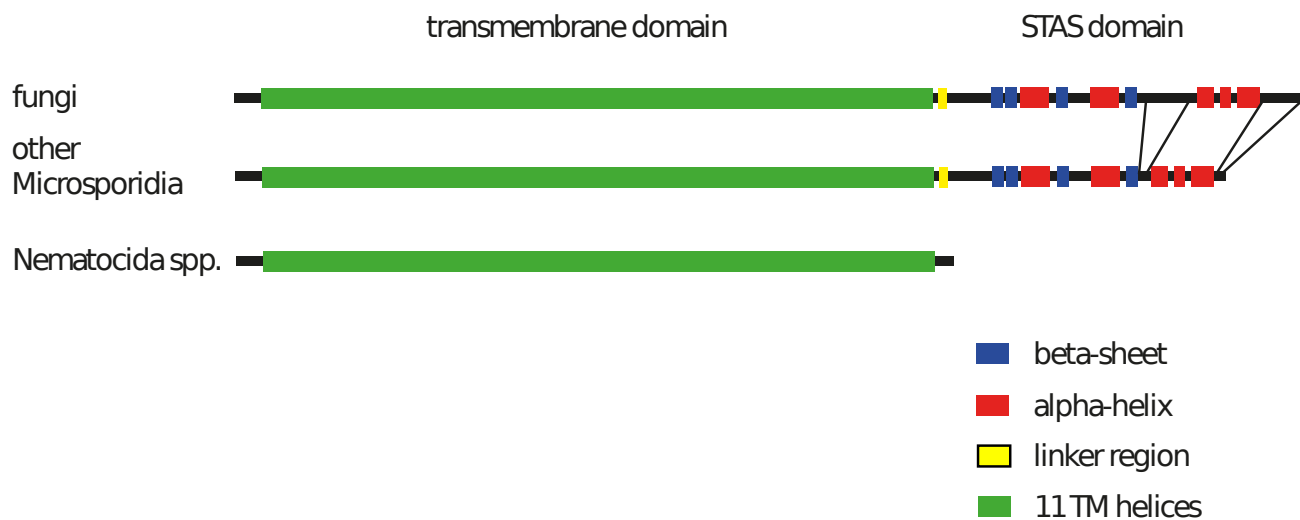

Figure S7

(a)

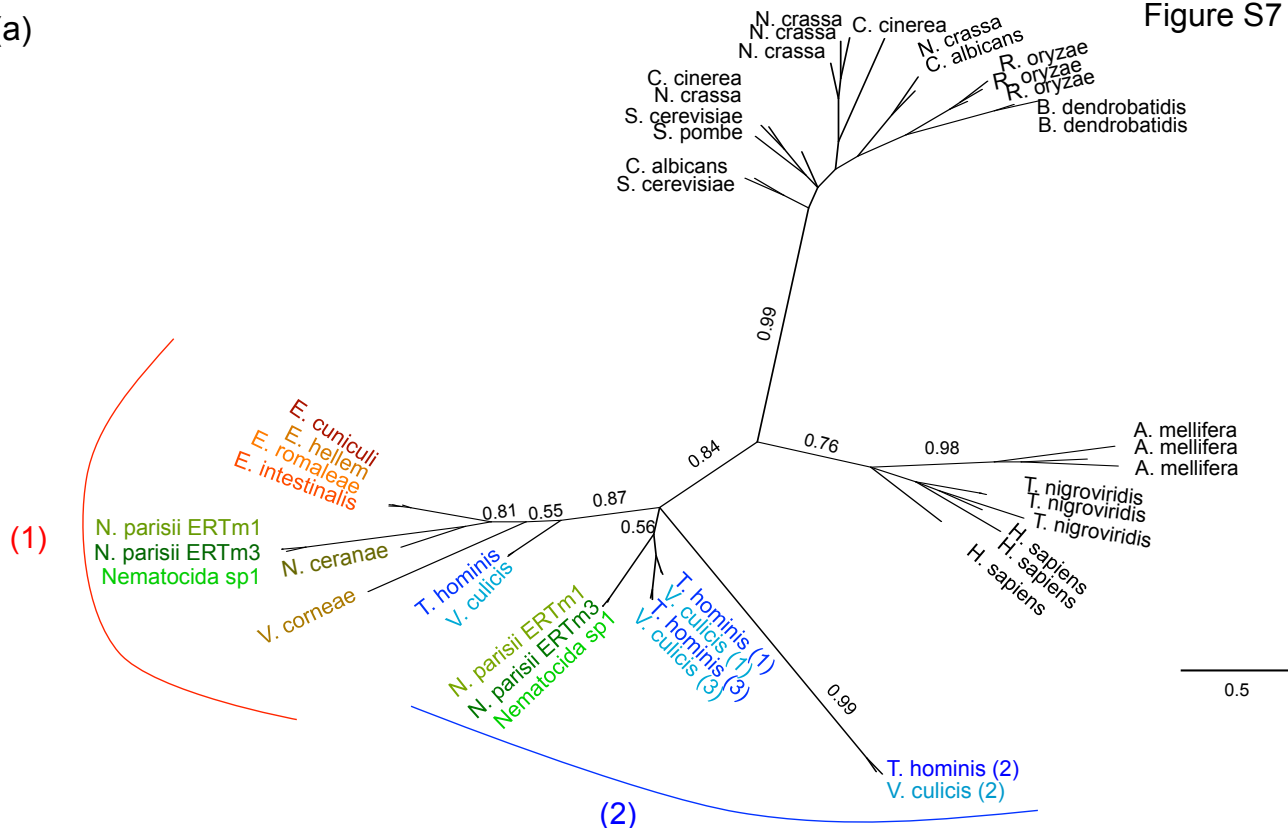

(b)

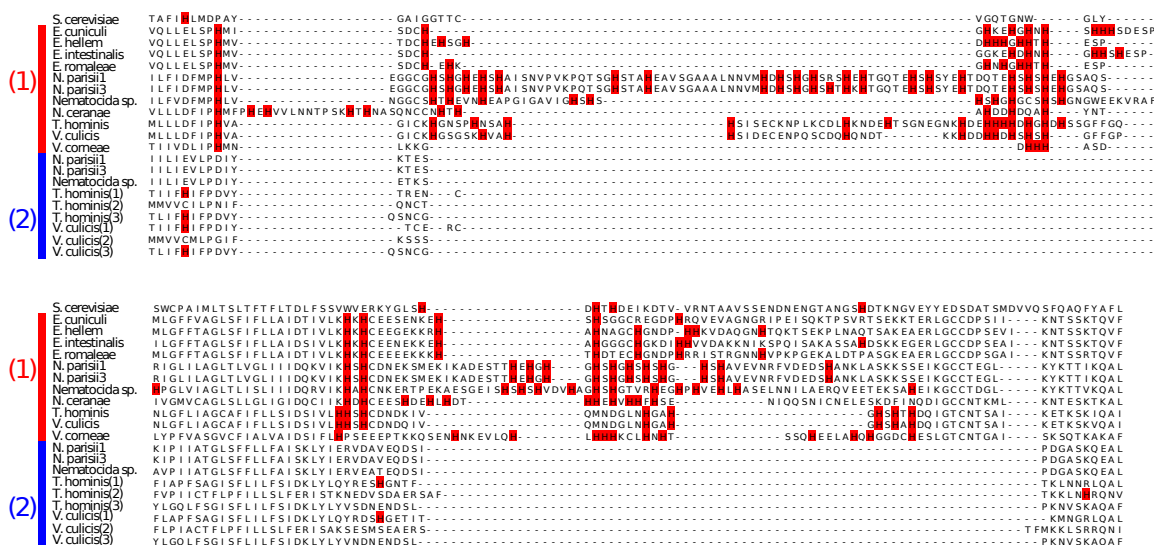

Figure S8

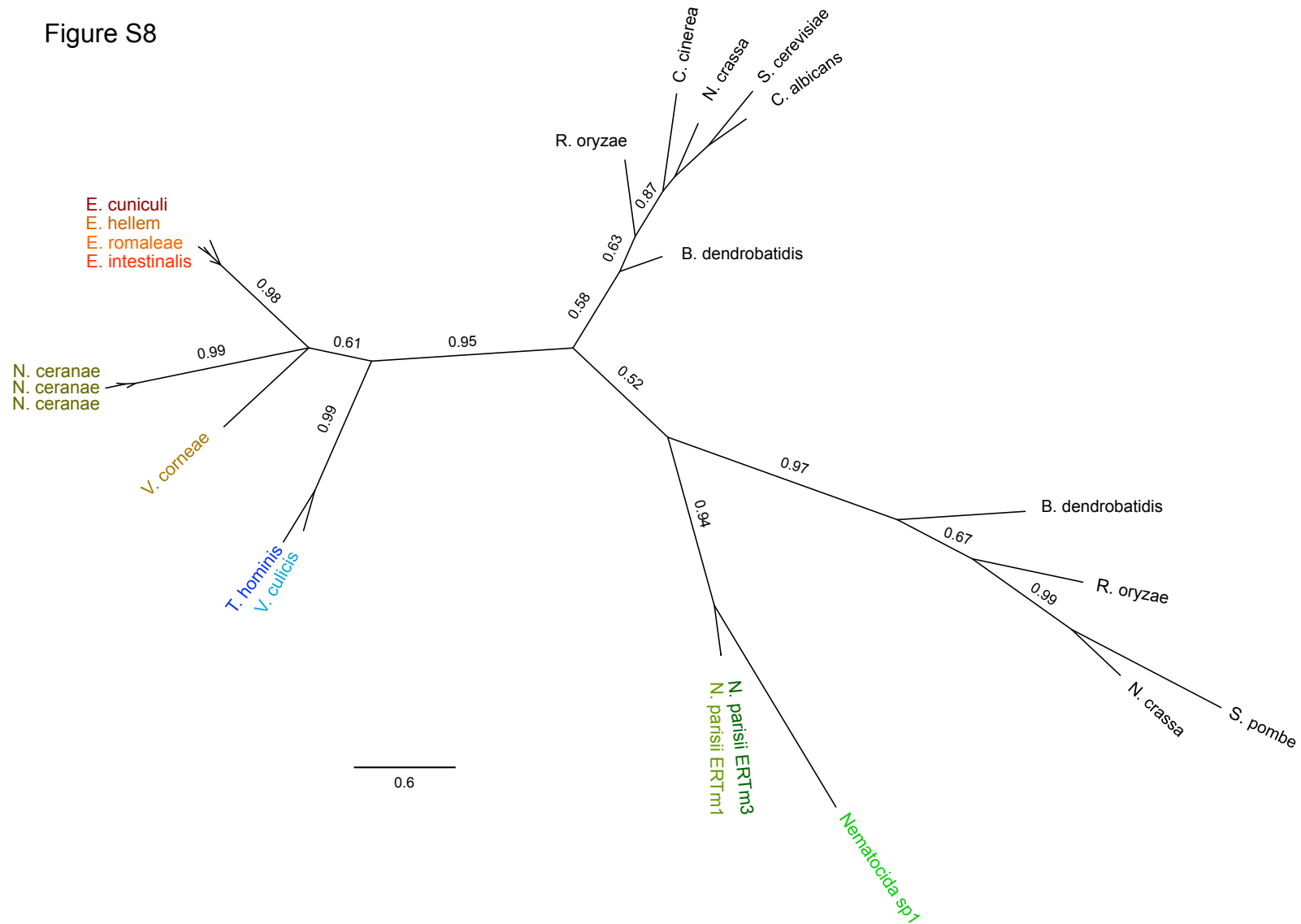

Figure S9

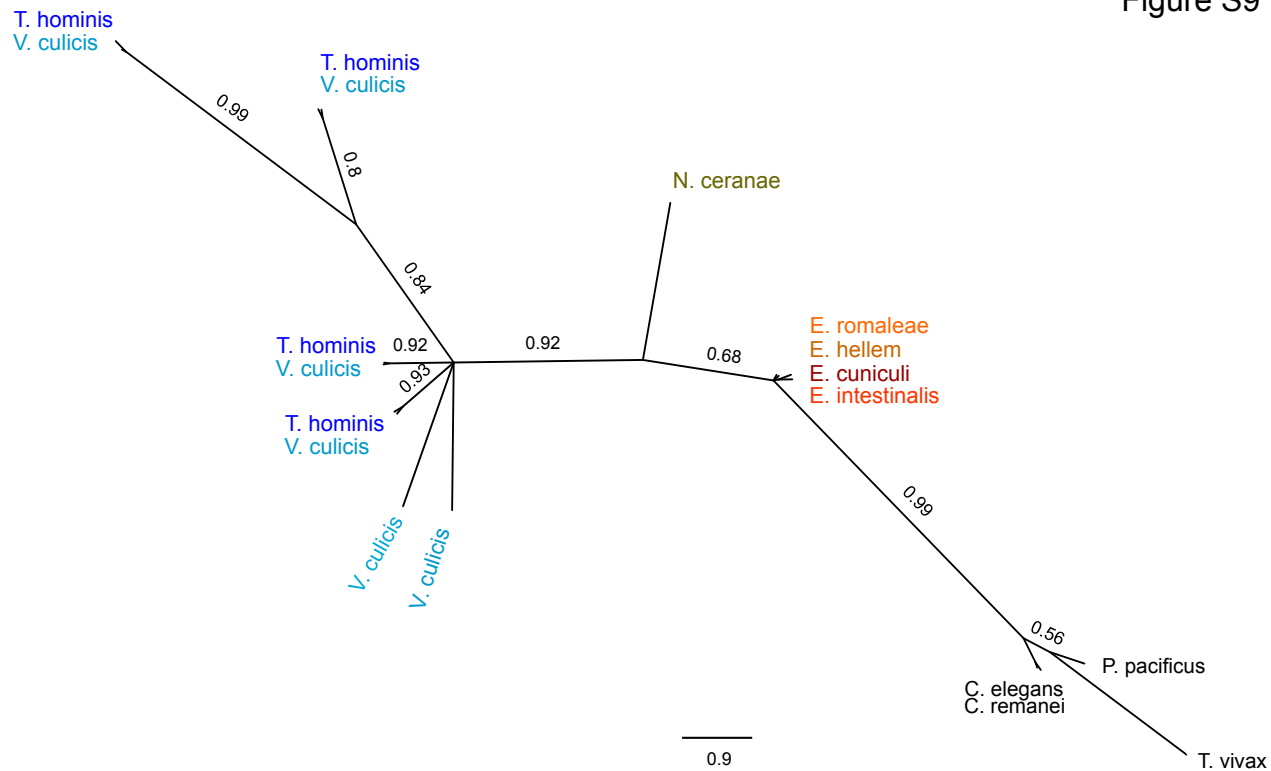

Figure S10

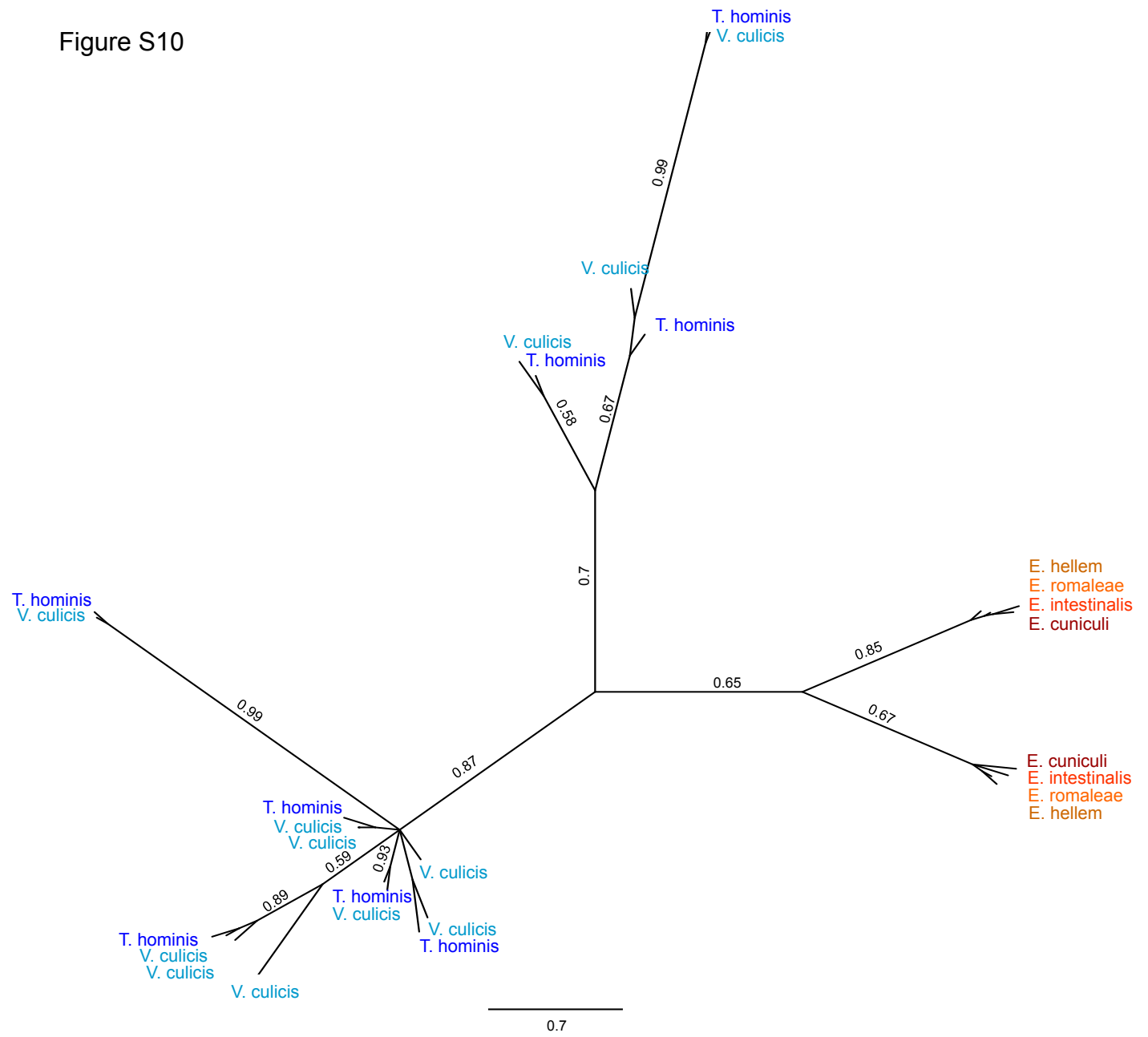

Supplement: Supplementary Data [file supp_evt184_Figure_S1-S10.pdf]
