# Supplementary material for: Reduction and Expansion in Microsporidian Genome Evolution: New Insights from Comparative Genomics
Source: Genome Biol Evol. 2013 Nov 19;5(12):2285–303. doi: 10.1093/gbe/evt184 (PMC3879972; doi:10.1093/gbe/evt184)
Supplement: Supplementary Data [file supp_evt184_Nakjang_etal_Supplementary_information.doc]

**Supplementary information**

**Fungal and animal genome sequence data**

The following fungal genomes were included in the analysis: *Saccharomyces cerevisiae* S288c (RefSeq: NC_001133-NC_001148, NC_001224); *Candida albicans* SC5314 (haploid genome version from the *Candida* Genome database , Assembly 21 (van het Hoog et al. 2007), downloaded June 13, 2012); *Schizosaccharomyces pombe* 972h (RefSeq: NC_003421-NC_003424, NC_001326); *Neurospora crassa* OR74A (RefSeq: NW_001849581-NW_001849831); *Coprinopsis cinerea* okayama7#130 (RefSeq: NW_003307477-NW_003307544); *Rhizopus oryzae* strain RA 99-880 (GenBank: CH476732-CH476759, GG669511-GG669565); and *Batrachochytrium dendrobatidis* JEL423 , downloaded August 3, 2012). Three animal genomes included in the analysis are: *Homo sapiens* (Venter JC et al. 2001)(<http://www.uniprot.org/>, Uniprot version containing one protein per gene; downloaded January 20, 2011); *Tetraodon nigroviridis* (GenBank: CAAE01000001-CAAE01025773); and *Apis mellifera* (RefSeq: NC_007070-NC007085, NC_001566).

**Supplementary tables and figures**

**Supplementary table S1** List of microsporidian genomes analyzed in this study.

**Supplementary table S2** Novel microsporidian-specific protein families. These are protein families that contain only homologues from Microsporidia, in that no homologues from outside the Microsporidia could be identified either by BLASTP or HHsearch. The presence of protein family members in published expression data are shown: 'yes' indicates present, 'no' denotes absence, and 'NA' indicates that no members of a given protein family are found in that species.

**Supplementary table S3** Microsporidia-specific core protein families. These families are conserved across at least 9 (out of 11) microsporidian genomes analyzed, but with no detectable homologues outside the Microsporidia. The results of N-terminal signal peptide and transmembrane domains predictions by SignalP 4.0 and TMHMM 2.0 respectively are given. The evidence for gene expression in published transcriptomic or proteomic data are also provided.

**Supplementary table S4** Core microsporidian protein families. These families are conserved across at least 9 microsporidian genomes (out of 11 analyzed in this study).

**Supplementary table S5** Protein families with microsporidian-specific gene duplications. We used gene trees to infer Microsporidia-specific duplications. Protein families were classified as expanded within the Microsporidia if the microsporidian sequences are monophyletic (with bootstrap support > 80%) and contain more than one microsporidian sequence from the same taxon, to the exclusion of sequences from outside the Microsporidia. For each family, the number of duplicates in each taxon is shown. The total number of paralogues found among microsporidians and the number of taxa for which duplicates were identified are provided.

**Supplementary figure S1** Horizontal gene transfer of a glycosyl transferase from bacteria to Nematocida. The phylogenetic tree suggests a horizontal gene transfer of a glycosyl transferase from bacteria into the ancestor of Nematocida species; these genes are not found in any other sequenced Microsporidian. Two sequences marked (A) in the tree are from Archaea that may also have obtained this gene from bacteria by HGT. Two out of three Nematocida glycosyl transferase genes have acquired an N-terminal signal peptide (marked with blue dots), raising the possibility that these genes are secreted into the host cell. The tree was inferred using the CAT60 model in PhyloBayes; branch lengths are proportional to the number of substitutions per site, as indicated by the scale bar.

**Supplementary table S6** Microsporidian CAP-containing proteins BLASTP hit results. Table shows a list of entries outside Microsporidia that have the highest similarity to microsporidian CAP-containing proteins by BLASTP searches. The *E. cuniculi* CAP protein (ECU03_0990) was used as a query sequence for the searches.

**Supplementary figure S2** Lineage-specific expansion and gain of secretory signals in microsporidian hexokinases. The phylogenetic tree indicates a single copy of hexokinase at the LMCA, followed by independent, lineage-specific duplications of hexokinase during the evolution of Microsporidia, fungi and animals. Some microsporidian hexokinase sequences, but no animal or fungal sequences, are predicted to encode an N-terminal signal peptide (SP+), suggesting that some of the microsporidian sequences may be secreted. The tree was inferred using the CAT60 model in PhyloBayes; branch lengths are proportional to the number of substitutions per site, as indicated by the scale bar.

**Supplementary table S7** List of protein families with an overrepresentation of N-terminal signal peptides among microsporidian homologues compared to fungal outgroups.

**Supplementary figure S3** Phylogenetic analysis of microsporidian zinc metalloprotease Ste24 homologues. The tree suggests multiple independent expansions of Ste24 in Microsporidia. A duplication in the LCMA was followed by the divergence of one copy and the loss of the canonical eukaryotic copy in the lineage leading to *Nematocida* spp. The tree topology indicates additional lineage-specific expansion of the divergent copy in *Nosema* and *Encephalitozoon*. The tree was inferred using the CAT60 model in PhyloBayes; branch lengths are proportional to the number of substitutions per site, as indicated by the scale bar.

**Supplementary figure S4** Phylogenetic analysis of microsporidian Hsp90 homologues. While only a single copy of Hsp90 is conserved in most microsporidian genomes, two copies are present in *T. hominis* and *V. culicis*. The phylogeny suggests a gene duplication in the lineage leading to these two species. The long branch leading to the second duplicate indicates that it is evolving more quickly than the copy conserved in all Microsporidia, and this duplicate has an N-terminal signal peptide in both species (denoted by a blue dot). The tree was inferred using the CAT60 model in PhyloBayes; branch lengths are proportional to the number of substitutions per site, as indicated by the scale bar.

**Supplementary table S8** Ancestor-derived transporter protein families that have undergone gene duplication during the radiation of Microsporidia. Subcellular localization was inferred based on similarity to transporters from model eukaryotes.

**Supplementary figure S5** Duplication of a member of the major facilitator superfamily (MFS) has led to distinct subfamilies in Microsporidia. (a) Phylogenetic analysis of microsporidian MFS genes indicates the presence of two sub-groups in this family. The relationship of microsporidian putative NupG genes to other MFS families is not resolved in our phylogeny. It is therefore unclear whether the Microsporidia MFS might also function as a nucleoside transporter as described for some bacterial NupG. The subgroup (1) is present in all microsporidian genomes analyzed and has undergone a duplication event in the lineage leading to *Encephalitozoon* spp., *V. corneae* and *N. ceranae*, with one copy subsequently lost from *N. ceranae*. The second subgroup (2) has undergone numerous lineage-specific duplication events and has been lost from the lineage leading to *Vittaforma* and *Encephalitozoon* spp. ‘*’ indicates entries that have a NupG domain as predicted by InterProScan. (b) An insertion between the 7th and 8th alpha helix is observed among the microsporidian sequences from subgroup (1). This insertion is not present in other eukaryotic homologues or in the second Microsporidia subgroup. The alignment is shaded in red to denote increasing hydrophobicity. The tree was inferred using the CAT60 model in PhyloBayes; branch lengths are proportional to the number of substitutions per site, as indicated by the scale bar.

**Supplementary figure S6** Phylogenetic analysis of Microsporidia sulfate permeases (SulP)-like proteins. (a) The tree topology implies a single copy at the microsporidian common ancestor, which subsequently underwent several independent lineage-specific duplications in the lineage leading to *T. hominis* and *V. culicis*, and the lineage leading to *Nematocida* species. One copy has then duplicated further in *Nematocida* sp. 1 ERT m2. (b) Domain analysis of the SulP family reveals different levels of reduction in the microsporidian SulP-like homologues. The majority of microsporidian proteins still contain the transmembrane region as well as all elements of the STAS (Sulfate Transporter Antagonist of anti-Sigma factor) domain, with only some regions being reduced in length as indicated. The STAS domain has been lost from the *Nematocida* homologs, suggesting functional divergence. The tree was inferred using the CAT60 model in PhyloBayes; branch lengths are proportional to the number of substitutions per site, as indicated by the scale bar.

**Supplementary figure S7** Phylogenetic analysis of microsporidian zinc transporter (ZIP)-like proteins. (a) The tree topology is consistent with the duplication of an ancestral single-copy gene at the base before the radiation of the Microsporidia. One copy has then lost from the lineage leading to *Nosema*, *Vittaforma* and *Encephalitozoon* spp., while further duplication events have taken place in the lineage leading to *T. hominis* and *V. culicis*. (b) These duplicates appear to have diverged in function: one group is found in all extant Microsporidia and has retained the HXHXH motif, a zinc-binding region in the yeast Zpt1 protein , while the other group has been lost in some lineages and has no HXHXH motif. The region of the alignment of the zinc-binding region in Zpt1 is shown, and histidine residues are highlighted in red. The yeast protein Zpt1 binds zinc via a HXHXH motif, and all Microsporidia sequences from the conserved group (group 1) show an enrichment of histidines with at least one HXHXH motif present in all taxa. In contrast, for the sequences from group 2, there has been a loss of the HXHXH motif, and no conserved histidine can be observed, indicating a loss of its canonical function. Phylogenetic analysis indicates that the reduced paralogous group has been lost from the lineage leading to *Nosema*, *Vittaforma* and *Encephalitozoon* spp, but has been amplified by additional gene duplications on the branch leading to *T. hominis* and *V. culicis*. The tree was inferred using the CAT60 model in PhyloBayes; branch lengths are proportional to the number of substitutions per site, as indicated by the scale bar.

**Supplementary figure S8** Phylogenetic analysis of microsporidian choline transporter (CTL)-like proteins. The phylogeny implies a single-copy gene in the microsporidian common ancestor, which was subsequently duplicated in the lineage leading to *N. ceranae*, resulting in three copies in that lineage. The microsporidian protein members in this family are related to the yeast Pns1. Although Pns1 in yeast does not have any reported choline transport function, the gene was shown to rescue a deletion mutant that was incapable of transporting choline in the electric ray, *Torpedo marmorata* and is upregulated during drug-induced cell wall stress in yeast . It is noteworthy that yeast has a canonical, highly efficient choline transporter (Hnm1p; ) that is absent in Microsporidia. It is therefore possible that microsporidian Pns1 may act to supply the parasites with choline, which can be used as a precursor for the biosynthesis of phosphatidylcholine. The tree was inferred using the CAT60 model in PhyloBayes; branch lengths are proportional to the number of substitutions per site, as indicated by the scale bar.

**Supplementary figure S9** Phylogenetic analysis of microsporidian drug/metabolite transporter (DMT)-like proteins. The phylogeny implies that a single copy of a DMT-related gene was present in the LCMA, which subsequently duplicated in the lineage leading to *T. hominis*/*V. culicis*, resulting in six copies in *V. culicis* and four in *T. hominis*. The tree was inferred using the CAT60 model in PhyloBayes; branch lengths are proportional to the number of substitutions per site, as indicated by the scale bar.

**Supplementary figure S10** Lineage-specific expansions of a putative transporter family. The phylogeny demonstrates that a family of putative transporters of unknown function has undergone selective expansion in several Microsporidia lineages; there was a duplication in the ancestor of *Encephalitozoon* spp. leading to two copies conserved in all four *Encephalitozoon* species, whereas twelve copies in *V. culicis* and eight copies in T. hominis originate from duplications at different stages in the evolution of these taxa. The tree was inferred using the CAT60 model in PhyloBayes; branch lengths are proportional to the number of substitutions per site, as indicated by the scale bar.

**References**

Arnaud MB et al. 2012. *Candida* Genome Database. http://www.candidagenome.org/ (accessed June 13, 2012).

Consortium HGS. 2006. Insights into social insects from the genome of the honeybee *Apis mellifera*. Nature 443:931-949.

Galagan JE et al. 2003. The genome sequence of the filamentous fungus *Neurospora crassa*. Nature. 422:859-868.

García R, Rodríguez-Peña JM, Bermejo C, Nombela C, Arroyo J. 2009. The high osmotic response and cell wall integrity pathways cooperate to regulate transcriptional responses to zymolyase-induced cell wall stress in *Saccharomyces cerevisiae*. J Biol Chem. 284:10901-10911.

Ma L-J et al. 2009. Genomic analysis of the basal lineage fungus *Rhizopus oryzae* reveals a whole-genome duplication. PLoS Genetics 5: e1000549.

Murakami Y, Philippsen P, Tettelin H, Oliver SG. 1996. Life with 6000 Genes. Science 274:563-567.

Nikawa J, Hosaka K, Tsukagoshi Y, Yamashita S. 1990. Primary structure of the yeast choline transport gene and regulation of its expression. J Biol Chem. 265:15996-16003.

O'Regan S et al. 2000. An electric lobe suppressor for a yeast choline transport mutation belongs to a new family of transporter-like proteins. Proc Natl Acad Sci U S A. 97:1835-1840.

Roest Crollius H et al. 2000. Characterization and repeat analysis of the compact genome of the freshwater pufferfish *Tetraodon nigroviridis*. Genome Res. 10:939-949.

Stajich JE et al. 2010. Insights into evolution of multicellular fungi from the assembled chromosomes of the mushroom *Coprinopsis cinerea* (*Coprinus cinereus*). Proc Natl Acad Sci U S A. 107:11889-11894.

van het Hoog et al. 2007. Assembly of the *Candida albicans* genome into sixteen supercontigs aligned on the eight chromosomes. Genome Biol. 8:R52.

Venter JC et al. 2001. The sequence of the Human genome. Science 291:1304-1351.

Wood V et al. 2002. The genome sequence of *Schizosaccharomyces pombe*. Nature 415:871-880.

Zhao H, Eide D. 1996. The yeast ZRT1 gene encodes the zinc transporter protein of a high-affinity uptake system induced by zinc limitation. Proc Natl Acad Sci U S A. 93:2454-2458.
